# Supplementary material for: Health impacts of wildfire-related air pollution in Brazil: a nationwide study of more than 2 million hospital admissions between 2008 and 2018
Source: Nat Commun. 2021 Nov 12;12:6555. doi: 10.1038/s41467-021-26822-7 (PMC8589982; doi:10.1038/s41467-021-26822-7)
Supplement: Supplementary file 1 — Supplementary information [file 41467_2021_26822_MOESM1_ESM.docx]

**Supplementary information**

**Health impacts of wildfire-related air pollution in Brazil: A nationwide study of more than 2 million hospital admissions between 2008 and 2018**

**Weeberb J. Requia**

(Corresponding Author)

School of Public Policy and Government, Fundação Getúlio Vargas

Brasília, Distrito Federal, Brazil

**Heresh Amini**

Department of Public Health, University of Copenhagen

Copenhagen, Denmark

**Rajarshi Mukherjee**

Department of Biostatistics, Harvard T.H. Chan School of Public Health

Boston, Massachusetts, United States

**Diane R. Gold**

Harvard T.H. Chan School of Public Health, Harvard University

Boston, Massachusetts, United States

Channing Division of Network Medicine, Department of Medicine, Brigham and Women’s Hospital and Harvard Medical School, Boston, Massachusetts, United States

**Joel D. Schwartz**

Department of Environmental Health, Harvard TH Chan School of Public Health

Boston, Massachusetts, United States

**Supplementary Figure 1**

**
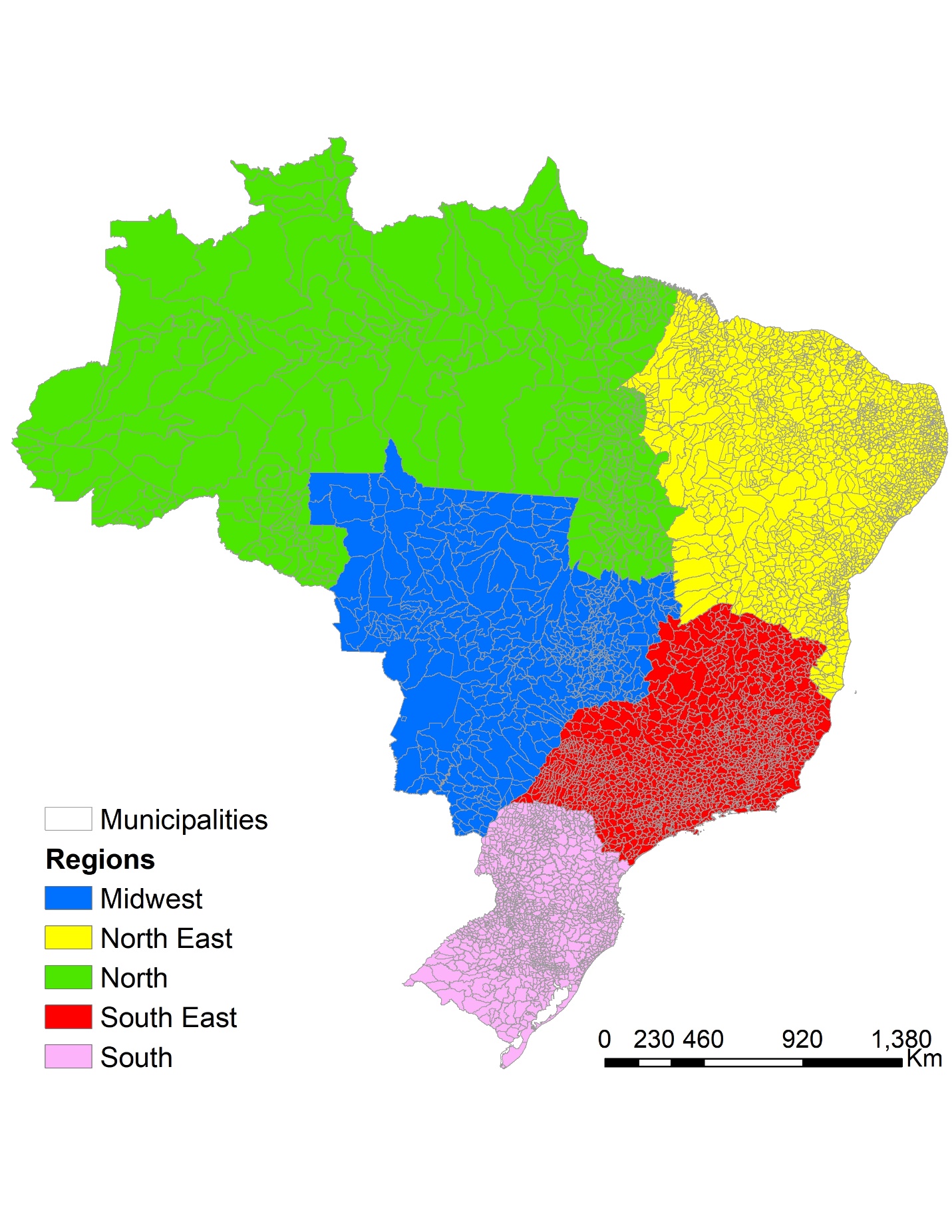
**

Spatial distribution of the municipalities and regions in Brazil.

**Supplementary Table**

Odds ratio and 95%CI for the Midwest region. Results are stratified by health outcome, model (primary and sensitivity analyses), and moving average (1-5). Note: “NA” means that there were very large groups of ties (a large number of events out of a large number of subjects). The algorithm in the Survival package may refuse to undertake the task (the computation is infeasible) due to overflow for the subscripts.

| Outcome | Model | Moving Average | Controls(n) | Cases(n) | Odds Ratio | Lower 95%CI | Upper 95%CI |
| --- | --- | --- | --- | --- | --- | --- | --- |
| Cardiorespiratory Hospital Admissions | Primary analysis | 1-day moving average | 147293 | 189 | 1.10 | 1.07 | 1.12 |
| Cardiorespiratory Hospital Admissions | Primary analysis | 2-days moving average | 147301 | 181 | 1.12 | 1.08 | 1.15 |
| Cardiorespiratory Hospital Admissions | Primary analysis | 3-days moving average | 147295 | 187 | 1.14 | 1.11 | 1.18 |
| Cardiorespiratory Hospital Admissions | Primary analysis | 4-days moving average | 147301 | 181 | 1.16 | 1.11 | 1.20 |
| Cardiorespiratory Hospital Admissions | Primary analysis | 5-days moving average | 147244 | 238 | 1.22 | 1.18 | 1.27 |
| Cardiorespiratory Hospital Admissions | Stratified by sex - Man | 1-day moving average | 77168 | 91 | 1.07 | 1.04 | 1.11 |
| Cardiorespiratory Hospital Admissions | Stratified by sex - Man | 2-days moving average | 77170 | 89 | 1.11 | 1.06 | 1.16 |
| Cardiorespiratory Hospital Admissions | Stratified by sex - Man | 3-days moving average | 77162 | 97 | 1.17 | 1.10 | 1.24 |
| Cardiorespiratory Hospital Admissions | Stratified by sex - Man | 4-days moving average | 77160 | 99 | 1.16 | 1.08 | 1.24 |
| Cardiorespiratory Hospital Admissions | Stratified by sex - Man | 5-days moving average | 77133 | 126 | 1.21 | 1.12 | 1.29 |
| Cardiorespiratory Hospital Admissions | Stratified by sex - Woman | 1-day moving average | 70133 | 90 | 1.12 | 1.07 | 1.16 |
| Cardiorespiratory Hospital Admissions | Stratified by sex - Woman | 2-days moving average | 70132 | 91 | 1.16 | 1.10 | 1.22 |
| Cardiorespiratory Hospital Admissions | Stratified by sex - Woman | 3-days moving average | 70133 | 90 | 1.12 | 1.07 | 1.18 |
| Cardiorespiratory Hospital Admissions | Stratified by sex - Woman | 4-days moving average | 70141 | 82 | 1.24 | 1.14 | 1.33 |
| Cardiorespiratory Hospital Admissions | Stratified by sex - Woman | 5-days moving average | 70114 | 109 | 1.44 | 1.33 | 1.56 |
| Cardiorespiratory Hospital Admissions | Stratified by age (0-5 years old) | 1-day moving average | NA | NA | NA | NA | NA |
| Cardiorespiratory Hospital Admissions | Stratified by age (0-5 years old) | 2-days moving average | NA | NA | NA | NA | NA |
| Cardiorespiratory Hospital Admissions | Stratified by age (0-5 years old) | 3-days moving average | NA | NA | NA | NA | NA |
| Cardiorespiratory Hospital Admissions | Stratified by age (0-5 years old) | 4-days moving average | NA | NA | NA | NA | NA |
| Cardiorespiratory Hospital Admissions | Stratified by age (0-5 years old) | 5-days moving average | 20429 | 40 | NA | NA | NA |
| Cardiorespiratory Hospital Admissions | Stratified by age (35-64 years old) | 1-day moving average | 49777 | 78 | 1.11 | 1.06 | 1.16 |
| Cardiorespiratory Hospital Admissions | Stratified by age (35-64 years old) | 2-days moving average | 49781 | 74 | 1.18 | 1.09 | 1.28 |
| Cardiorespiratory Hospital Admissions | Stratified by age (35-64 years old) | 3-days moving average | 49781 | 74 | 1.13 | 1.03 | 1.22 |
| Cardiorespiratory Hospital Admissions | Stratified by age (35-64 years old) | 4-days moving average | 49783 | 72 | 1.28 | 1.05 | 1.51 |
| Cardiorespiratory Hospital Admissions | Stratified by age (35-64 years old) | 5-days moving average | 49762 | 93 | 1.56 | 1.35 | 1.76 |
| Cardiorespiratory Hospital Admissions | Stratified by age (> 64 years old) | 1-day moving average | 51594 | 54 | 1.10 | 1.04 | 1.16 |
| Cardiorespiratory Hospital Admissions | Stratified by age (> 64 years old) | 2-days moving average | 51587 | 61 | 1.36 | 1.20 | 1.51 |
| Cardiorespiratory Hospital Admissions | Stratified by age (> 64 years old) | 3-days moving average | 51578 | 70 | 1.27 | 1.09 | 1.45 |
| Cardiorespiratory Hospital Admissions | Stratified by age (> 64 years old) | 4-days moving average | 51588 | 60 | 1.34 | 1.17 | 1.50 |
| Cardiorespiratory Hospital Admissions | Stratified by age (> 64 years old) | 5-days moving average | 51577 | 71 | 1.37 | 1.22 | 1.52 |
| Cardiorespiratory Hospital Admissions | Primary model excluding the other pollutants (CO, NO2, and O3) | 1-day moving average | 147293 | 189 | 1.19 | 1.17 | 1.21 |
| Cardiorespiratory Hospital Admissions | Primary model excluding the other pollutants (CO, NO2, and O3) | 2-days moving average | 147301 | 181 | 1.23 | 1.20 | 1.25 |
| Cardiorespiratory Hospital Admissions | Primary model excluding the other pollutants (CO, NO2, and O3) | 3-days moving average | 147295 | 187 | 1.26 | 1.23 | 1.29 |
| Cardiorespiratory Hospital Admissions | Primary model excluding the other pollutants (CO, NO2, and O3) | 4-days moving average | 147301 | 181 | 1.28 | 1.25 | 1.31 |
| Cardiorespiratory Hospital Admissions | Primary model excluding the other pollutants (CO, NO2, and O3) | 5-days moving average | 147244 | 238 | 1.34 | 1.30 | 1.37 |
| Cardiorespiratory Hospital Admissions | Primary model excluding race | 1-day moving average | 147293 | 189 | 1.10 | 1.07 | 1.12 |
| Cardiorespiratory Hospital Admissions | Primary model excluding race | 2-days moving average | 147301 | 181 | 1.12 | 1.08 | 1.15 |
| Cardiorespiratory Hospital Admissions | Primary model excluding race | 3-days moving average | 147295 | 187 | 1.14 | 1.10 | 1.18 |
| Cardiorespiratory Hospital Admissions | Primary model excluding race | 4-days moving average | 147301 | 181 | 1.16 | 1.11 | 1.20 |
| Cardiorespiratory Hospital Admissions | Primary model excluding race | 5-days moving average | 147244 | 238 | 1.22 | 1.17 | 1.27 |
| Cardiorespiratory Hospital Admissions | Primary model excluding state and lat/long | 1-day moving average | 147293 | 189 | 1.10 | 1.07 | 1.12 |
| Cardiorespiratory Hospital Admissions | Primary model excluding state and lat/long | 2-days moving average | 147301 | 181 | 1.12 | 1.09 | 1.15 |
| Cardiorespiratory Hospital Admissions | Primary model excluding state and lat/long | 3-days moving average | 147295 | 187 | 1.15 | 1.11 | 1.18 |
| Cardiorespiratory Hospital Admissions | Primary model excluding state and lat/long | 4-days moving average | 147301 | 181 | 1.16 | 1.12 | 1.20 |
| Cardiorespiratory Hospital Admissions | Primary model excluding state and lat/long | 5-days moving average | 147244 | 238 | 1.23 | 1.18 | 1.28 |
| Circulatory Hospital Admissions | Primary analysis | 1-day moving average | 67840 | 100 | 1.09 | 1.06 | 1.13 |
| Circulatory Hospital Admissions | Primary analysis | 2-days moving average | 67850 | 90 | 1.56 | 1.36 | 1.77 |
| Circulatory Hospital Admissions | Primary analysis | 3-days moving average | 67844 | 96 | 1.19 | 1.10 | 1.27 |
| Circulatory Hospital Admissions | Primary analysis | 4-days moving average | 67844 | 96 | 1.20 | 1.12 | 1.28 |
| Circulatory Hospital Admissions | Primary analysis | 5-days moving average | 67828 | 112 | 1.33 | 1.23 | 1.44 |
| Circulatory Hospital Admissions | Stratified by sex - Man | 1-day moving average | 34971 | 50 | 1.30 | 1.30 | 1.30 |
| Circulatory Hospital Admissions | Stratified by sex - Man | 2-days moving average | NA | NA | NA | NA | NA |
| Circulatory Hospital Admissions | Stratified by sex - Man | 3-days moving average | 34967 | 54 | 6.87 | 6.87 | 6.87 |
| Circulatory Hospital Admissions | Stratified by sex - Man | 4-days moving average | 34964 | 57 | NA | NA | NA |
| Circulatory Hospital Admissions | Stratified by sex - Man | 5-days moving average | 34959 | 62 | NA | NA | NA |
| Circulatory Hospital Admissions | Stratified by sex - Woman | 1-day moving average | 32874 | 45 | 11.90 | 11.90 | 11.90 |
| Circulatory Hospital Admissions | Stratified by sex - Woman | 2-days moving average | 32874 | 45 | 38.95 | 38.95 | 38.95 |
| Circulatory Hospital Admissions | Stratified by sex - Woman | 3-days moving average | NA | NA | NA | NA | NA |
| Circulatory Hospital Admissions | Stratified by sex - Woman | 4-days moving average | 32880 | 39 | 1.60 | 0.89 | 2.31 |
| Circulatory Hospital Admissions | Stratified by sex - Woman | 5-days moving average | 32872 | 47 | 9.06 | 9.06 | 9.06 |
| Circulatory Hospital Admissions | Stratified by age (0-5 years old) | 1-day moving average | 663 | NA | NA | NA | NA |
| Circulatory Hospital Admissions | Stratified by age (0-5 years old) | 2-days moving average | 663 | NA | NA | NA | NA |
| Circulatory Hospital Admissions | Stratified by age (0-5 years old) | 3-days moving average | 663 | NA | NA | NA | NA |
| Circulatory Hospital Admissions | Stratified by age (0-5 years old) | 4-days moving average | 663 | NA | NA | NA | NA |
| Circulatory Hospital Admissions | Stratified by age (0-5 years old) | 5-days moving average | NA | NA | NA | NA | NA |
| Circulatory Hospital Admissions | Stratified by age (35-64 years old) | 1-day moving average | 32427 | 57 | 1.13 | 1.05 | 1.21 |
| Circulatory Hospital Admissions | Stratified by age (35-64 years old) | 2-days moving average | NA | NA | NA | NA | NA |
| Circulatory Hospital Admissions | Stratified by age (35-64 years old) | 3-days moving average | NA | NA | NA | NA | NA |
| Circulatory Hospital Admissions | Stratified by age (35-64 years old) | 4-days moving average | 32432 | 52 | 1.41 | 0.89 | 1.94 |
| Circulatory Hospital Admissions | Stratified by age (35-64 years old) | 5-days moving average | NA | NA | NA | NA | NA |
| Circulatory Hospital Admissions | Stratified by age (> 64 years old) | 1-day moving average | 29349 | 37 | 1.11 | 1.01 | 1.21 |
| Circulatory Hospital Admissions | Stratified by age (> 64 years old) | 2-days moving average | NA | NA | NA | NA | NA |
| Circulatory Hospital Admissions | Stratified by age (> 64 years old) | 3-days moving average | NA | NA | NA | NA | NA |
| Circulatory Hospital Admissions | Stratified by age (> 64 years old) | 4-days moving average | NA | NA | NA | NA | NA |
| Circulatory Hospital Admissions | Stratified by age (> 64 years old) | 5-days moving average | NA | NA | NA | NA | NA |
| Circulatory Hospital Admissions | Primary model excluding the other pollutants (CO, NO2, and O3) | 1-day moving average | 67840 | 100 | 1.20 | 1.17 | 1.23 |
| Circulatory Hospital Admissions | Primary model excluding the other pollutants (CO, NO2, and O3) | 2-days moving average | 67850 | 90 | 1.42 | 1.32 | 1.51 |
| Circulatory Hospital Admissions | Primary model excluding the other pollutants (CO, NO2, and O3) | 3-days moving average | 67844 | 96 | 1.31 | 1.25 | 1.37 |
| Circulatory Hospital Admissions | Primary model excluding the other pollutants (CO, NO2, and O3) | 4-days moving average | 67844 | 96 | 1.34 | 1.27 | 1.41 |
| Circulatory Hospital Admissions | Primary model excluding the other pollutants (CO, NO2, and O3) | 5-days moving average | 67828 | 112 | 1.45 | 1.36 | 1.55 |
| Circulatory Hospital Admissions | Primary model excluding race | 1-day moving average | 67840 | 100 | 1.09 | 1.06 | 1.13 |
| Circulatory Hospital Admissions | Primary model excluding race | 2-days moving average | 67850 | 90 | 1.49 | 1.31 | 1.67 |
| Circulatory Hospital Admissions | Primary model excluding race | 3-days moving average | 67844 | 96 | 1.17 | 1.10 | 1.25 |
| Circulatory Hospital Admissions | Primary model excluding race | 4-days moving average | 67844 | 96 | 1.20 | 1.12 | 1.28 |
| Circulatory Hospital Admissions | Primary model excluding race | 5-days moving average | 67828 | 112 | 1.30 | 1.21 | 1.39 |
| Circulatory Hospital Admissions | Primary model excluding state and lat/long | 1-day moving average | 67840 | 100 | 1.10 | 1.06 | 1.13 |
| Circulatory Hospital Admissions | Primary model excluding state and lat/long | 2-days moving average | 67850 | 90 | 1.37 | 1.26 | 1.49 |
| Circulatory Hospital Admissions | Primary model excluding state and lat/long | 3-days moving average | 67844 | 96 | 1.16 | 1.09 | 1.23 |
| Circulatory Hospital Admissions | Primary model excluding state and lat/long | 4-days moving average | 67844 | 96 | 1.20 | 1.13 | 1.27 |
| Circulatory Hospital Admissions | Primary model excluding state and lat/long | 5-days moving average | 67828 | 112 | 1.35 | 1.25 | 1.45 |
| Respiratory Hospital Admissions | Primary analysis | 1-day moving average | 79441 | 101 | 1.12 | 1.08 | 1.17 |
| Respiratory Hospital Admissions | Primary analysis | 2-days moving average | 79451 | 91 | 1.06 | 1.02 | 1.10 |
| Respiratory Hospital Admissions | Primary analysis | 3-days moving average | 79449 | 93 | 1.15 | 1.09 | 1.21 |
| Respiratory Hospital Admissions | Primary analysis | 4-days moving average | 79451 | 91 | 1.16 | 1.09 | 1.23 |
| Respiratory Hospital Admissions | Primary analysis | 5-days moving average | 79403 | 139 | 1.20 | 1.14 | 1.26 |
| Respiratory Hospital Admissions | Stratified by sex - Man | 1-day moving average | 42192 | 46 | NA | NA | NA |
| Respiratory Hospital Admissions | Stratified by sex - Man | 2-days moving average | 42195 | 43 | NA | NA | NA |
| Respiratory Hospital Admissions | Stratified by sex - Man | 3-days moving average | 42192 | 46 | 1.34 | 1.16 | 1.52 |
| Respiratory Hospital Admissions | Stratified by sex - Man | 4-days moving average | 42193 | 45 | 1.32 | 1.11 | 1.53 |
| Respiratory Hospital Admissions | Stratified by sex - Man | 5-days moving average | 42167 | 71 | 1.38 | 1.15 | 1.60 |
| Respiratory Hospital Admissions | Stratified by sex - Woman | 1-day moving average | 37252 | 52 | 1.06 | 1.03 | 1.10 |
| Respiratory Hospital Admissions | Stratified by sex - Woman | 2-days moving average | 37256 | 48 | 1.10 | 1.01 | 1.19 |
| Respiratory Hospital Admissions | Stratified by sex - Woman | 3-days moving average | 37257 | 47 | 1.23 | 1.07 | 1.38 |
| Respiratory Hospital Admissions | Stratified by sex - Woman | 4-days moving average | 37261 | 43 | NA | NA | NA |
| Respiratory Hospital Admissions | Stratified by sex - Woman | 5-days moving average | 37236 | 68 | 1.29 | 1.07 | 1.52 |
| Respiratory Hospital Admissions | Stratified by age (0-5 years old) | 1-day moving average | 19782 | 24 | NA | NA | NA |
| Respiratory Hospital Admissions | Stratified by age (0-5 years old) | 2-days moving average | 19785 | 21 | NA | NA | NA |
| Respiratory Hospital Admissions | Stratified by age (0-5 years old) | 3-days moving average | NA | NA | NA | NA | NA |
| Respiratory Hospital Admissions | Stratified by age (0-5 years old) | 4-days moving average | 19783 | 23 | NA | NA | NA |
| Respiratory Hospital Admissions | Stratified by age (0-5 years old) | 5-days moving average | NA | NA | NA | NA | NA |
| Respiratory Hospital Admissions | Stratified by age (35-64 years old) | 1-day moving average | 17349 | 22 | NA | NA | NA |
| Respiratory Hospital Admissions | Stratified by age (35-64 years old) | 2-days moving average | 17341 | 30 | NA | NA | NA |
| Respiratory Hospital Admissions | Stratified by age (35-64 years old) | 3-days moving average | 17346 | 25 | NA | NA | NA |
| Respiratory Hospital Admissions | Stratified by age (35-64 years old) | 4-days moving average | 17351 | 20 | NA | NA | NA |
| Respiratory Hospital Admissions | Stratified by age (35-64 years old) | 5-days moving average | 17339 | 32 | NA | NA | NA |
| Respiratory Hospital Admissions | Stratified by age (> 64 years old) | 1-day moving average | 22240 | 22 | NA | NA | NA |
| Respiratory Hospital Admissions | Stratified by age (> 64 years old) | 2-days moving average | 22239 | 23 | NA | NA | NA |
| Respiratory Hospital Admissions | Stratified by age (> 64 years old) | 3-days moving average | 22233 | 29 | NA | NA | NA |
| Respiratory Hospital Admissions | Stratified by age (> 64 years old) | 4-days moving average | 22238 | 24 | NA | NA | NA |
| Respiratory Hospital Admissions | Stratified by age (> 64 years old) | 5-days moving average | 22230 | 32 | NA | NA | NA |
| Respiratory Hospital Admissions | Primary model excluding the other pollutants (CO, NO2, and O3) | 1-day moving average | 79441 | 101 | 1.22 | 1.19 | 1.25 |
| Respiratory Hospital Admissions | Primary model excluding the other pollutants (CO, NO2, and O3) | 2-days moving average | 79451 | 91 | 1.19 | 1.16 | 1.22 |
| Respiratory Hospital Admissions | Primary model excluding the other pollutants (CO, NO2, and O3) | 3-days moving average | 79449 | 93 | 1.27 | 1.22 | 1.31 |
| Respiratory Hospital Admissions | Primary model excluding the other pollutants (CO, NO2, and O3) | 4-days moving average | 79451 | 91 | 1.29 | 1.24 | 1.35 |
| Respiratory Hospital Admissions | Primary model excluding the other pollutants (CO, NO2, and O3) | 5-days moving average | 79403 | 139 | 1.34 | 1.29 | 1.39 |
| Respiratory Hospital Admissions | Primary model excluding race | 1-day moving average | 79441 | 101 | 1.12 | 1.08 | 1.16 |
| Respiratory Hospital Admissions | Primary model excluding race | 2-days moving average | 79451 | 91 | 1.06 | 1.02 | 1.10 |
| Respiratory Hospital Admissions | Primary model excluding race | 3-days moving average | 79449 | 93 | 1.14 | 1.08 | 1.19 |
| Respiratory Hospital Admissions | Primary model excluding race | 4-days moving average | 79451 | 91 | 1.16 | 1.09 | 1.23 |
| Respiratory Hospital Admissions | Primary model excluding race | 5-days moving average | 79403 | 139 | 1.20 | 1.14 | 1.25 |
| Respiratory Hospital Admissions | Primary model excluding state and lat/long | 1-day moving average | 79441 | 101 | 1.12 | 1.08 | 1.16 |
| Respiratory Hospital Admissions | Primary model excluding state and lat/long | 2-days moving average | 79451 | 91 | 1.07 | 1.03 | 1.11 |
| Respiratory Hospital Admissions | Primary model excluding state and lat/long | 3-days moving average | 79449 | 93 | 1.15 | 1.09 | 1.21 |
| Respiratory Hospital Admissions | Primary model excluding state and lat/long | 4-days moving average | 79451 | 91 | 1.17 | 1.10 | 1.24 |
| Respiratory Hospital Admissions | Primary model excluding state and lat/long | 5-days moving average | 79403 | 139 | 1.20 | 1.14 | 1.25 |

Table 2 (Appendix 2): Odds ratio and 95%CI for the North East region. Results are stratified by health outcome, model (primary and sensitivity analyses), moving average (1-5). Note: “NA” means that there were very large groups of ties (a large number of events out of a large number of subjects). The algorithm in the Survival package may refuse to undertake the task (the computation is infeasible) due to overflow for the subscripts.

| Outcome | Model | Moving Average | Controls(n) | Cases(n) | Odds Ratio | Lower 95%CI | Upper 95%CI |
| --- | --- | --- | --- | --- | --- | --- | --- |
| Cardiorespiratory Hospital Admissions | Primary analysis | 1-day moving average | 496195 | 419 | 1.11 | 1.08 | 1.14 |
| Cardiorespiratory Hospital Admissions | Primary analysis | 2-days moving average | 496148 | 466 | 1.14 | 1.11 | 1.17 |
| Cardiorespiratory Hospital Admissions | Primary analysis | 3-days moving average | 496104 | 510 | 1.14 | 1.11 | 1.17 |
| Cardiorespiratory Hospital Admissions | Primary analysis | 4-days moving average | NA | NA | NA | NA | NA |
| Cardiorespiratory Hospital Admissions | Primary analysis | 5-days moving average | NA | NA | NA | NA | NA |
| Cardiorespiratory Hospital Admissions | Stratified by sex - Man | 1-day moving average | 250294 | 590 | 1.10 | 1.08 | 1.12 |
| Cardiorespiratory Hospital Admissions | Stratified by sex - Man | 2-days moving average | 250257 | 627 | 1.13 | 1.10 | 1.16 |
| Cardiorespiratory Hospital Admissions | Stratified by sex - Man | 3-days moving average | 250253 | 631 | 1.15 | 1.12 | 1.18 |
| Cardiorespiratory Hospital Admissions | Stratified by sex - Man | 4-days moving average | 250241 | 643 | NA | NA | NA |
| Cardiorespiratory Hospital Admissions | Stratified by sex - Man | 5-days moving average | 250226 | 658 | NA | NA | NA |
| Cardiorespiratory Hospital Admissions | Stratified by sex - Woman | 1-day moving average | 245168 | 562 | 1.09 | 1.06 | 1.11 |
| Cardiorespiratory Hospital Admissions | Stratified by sex - Woman | 2-days moving average | 245101 | 629 | 1.12 | 1.09 | 1.14 |
| Cardiorespiratory Hospital Admissions | Stratified by sex - Woman | 3-days moving average | 245068 | 662 | 1.15 | 1.12 | 1.17 |
| Cardiorespiratory Hospital Admissions | Stratified by sex - Woman | 4-days moving average | 245085 | 645 | NA | NA | NA |
| Cardiorespiratory Hospital Admissions | Stratified by sex - Woman | 5-days moving average | NA | NA | NA | NA | NA |
| Cardiorespiratory Hospital Admissions | Stratified by age (0-5 years old) | 1-day moving average | 84409 | 225 | 1.12 | 1.08 | 1.16 |
| Cardiorespiratory Hospital Admissions | Stratified by age (0-5 years old) | 2-days moving average | 84433 | 201 | 1.13 | 1.08 | 1.18 |
| Cardiorespiratory Hospital Admissions | Stratified by age (0-5 years old) | 3-days moving average | 84398 | 236 | 1.19 | 1.14 | 1.25 |
| Cardiorespiratory Hospital Admissions | Stratified by age (0-5 years old) | 4-days moving average | 84414 | 220 | 1.24 | 1.19 | 1.30 |
| Cardiorespiratory Hospital Admissions | Stratified by age (0-5 years old) | 5-days moving average | 84401 | 233 | 1.18 | 1.13 | 1.24 |
| Circulatory Hospital Admissions | Primary analysis | 1-day moving average | 223733 | 554 | 1.10 | 1.08 | 1.12 |
| Circulatory Hospital Admissions | Primary analysis | 2-days moving average | 223788 | 499 | 1.10 | 1.07 | 1.12 |
| Circulatory Hospital Admissions | Primary analysis | 3-days moving average | 223675 | 612 | 1.10 | 1.07 | 1.13 |
| Circulatory Hospital Admissions | Primary analysis | 4-days moving average | 223726 | 561 | 1.11 | 1.08 | 1.14 |
| Circulatory Hospital Admissions | Primary analysis | 5-days moving average | 223642 | 645 | 1.13 | 1.10 | 1.16 |
| Circulatory Hospital Admissions | Stratified by sex - Man | 1-day moving average | 109539 | 268 | 1.10 | 1.07 | 1.13 |
| Circulatory Hospital Admissions | Stratified by sex - Man | 2-days moving average | 109572 | 235 | 0.00 | 0.00 | 0.00 |
| Circulatory Hospital Admissions | Stratified by sex - Man | 3-days moving average | NA | NA | NA | NA | NA |
| Circulatory Hospital Admissions | Stratified by sex - Man | 4-days moving average | NA | NA | NA | NA | NA |
| Circulatory Hospital Admissions | Stratified by sex - Man | 5-days moving average | NA | NA | NA | NA | NA |
| Circulatory Hospital Admissions | Stratified by sex - Woman | 1-day moving average | 114375 | 105 | 1.15 | 1.09 | 1.22 |
| Circulatory Hospital Admissions | Stratified by sex - Woman | 2-days moving average | NA | NA | NA | NA | NA |
| Circulatory Hospital Admissions | Stratified by sex - Woman | 3-days moving average | 114360 | 120 | 0.00 | 0.00 | 0.00 |
| Circulatory Hospital Admissions | Stratified by sex - Woman | 4-days moving average | NA | NA | NA | NA | NA |
| Circulatory Hospital Admissions | Stratified by sex - Woman | 5-days moving average | NA | NA | NA | NA | NA |
| Circulatory Hospital Admissions | Stratified by age (0-5 years old) | 1-day moving average | NA | NA | NA | NA | NA |
| Circulatory Hospital Admissions | Stratified by age (0-5 years old) | 2-days moving average | NA | NA | NA | NA | NA |
| Circulatory Hospital Admissions | Stratified by age (0-5 years old) | 3-days moving average | NA | NA | NA | NA | NA |
| Circulatory Hospital Admissions | Stratified by age (0-5 years old) | 4-days moving average | NA | NA | NA | NA | NA |
| Circulatory Hospital Admissions | Stratified by age (0-5 years old) | 5-days moving average | NA | NA | NA | NA | NA |
| Circulatory Hospital Admissions | Stratified by age (35-64 years old) | 1-day moving average | 95752 | 226 | 0.00 | 0.00 | 0.00 |
| Circulatory Hospital Admissions | Stratified by age (35-64 years old) | 2-days moving average | NA | NA | NA | NA | NA |
| Circulatory Hospital Admissions | Stratified by age (35-64 years old) | 3-days moving average | 95720 | 258 | 1.12 | 1.07 | 1.17 |
| Circulatory Hospital Admissions | Stratified by age (35-64 years old) | 4-days moving average | 95745 | 233 | 1.10 | 1.06 | 1.15 |
| Circulatory Hospital Admissions | Stratified by age (35-64 years old) | 5-days moving average | 95703 | 275 | 1.13 | 1.08 | 1.19 |
| Circulatory Hospital Admissions | Stratified by age (> 64 years old) | 1-day moving average | 106046 | 261 | 1.08 | 1.04 | 1.11 |
| Circulatory Hospital Admissions | Stratified by age (> 64 years old) | 2-days moving average | 106063 | 244 | 1.09 | 1.05 | 1.13 |
| Circulatory Hospital Admissions | Stratified by age (> 64 years old) | 3-days moving average | 106020 | 287 | 1.07 | 1.04 | 1.11 |
| Circulatory Hospital Admissions | Stratified by age (> 64 years old) | 4-days moving average | 106048 | 259 | 1.11 | 1.07 | 1.16 |
| Circulatory Hospital Admissions | Stratified by age (> 64 years old) | 5-days moving average | 106016 | 291 | 1.10 | 1.06 | 1.15 |
| Circulatory Hospital Admissions | Primary model excluding the other pollutants (CO, NO2, and O3) | 1-day moving average | 223733 | 554 | 1.18 | 1.17 | 1.19 |
| Circulatory Hospital Admissions | Primary model excluding the other pollutants (CO, NO2, and O3) | 2-days moving average | 223788 | 499 | 1.19 | 1.18 | 1.20 |
| Circulatory Hospital Admissions | Primary model excluding the other pollutants (CO, NO2, and O3) | 3-days moving average | 223675 | 612 | 1.22 | 1.21 | 1.23 |
| Circulatory Hospital Admissions | Primary model excluding the other pollutants (CO, NO2, and O3) | 4-days moving average | 223726 | 561 | 1.22 | 1.21 | 1.23 |
| Circulatory Hospital Admissions | Primary model excluding the other pollutants (CO, NO2, and O3) | 5-days moving average | 223642 | 645 | 1.25 | 1.23 | 1.26 |
| Circulatory Hospital Admissions | Primary model excluding race | 1-day moving average | 223733 | 554 | 1.10 | 1.08 | 1.12 |
| Circulatory Hospital Admissions | Primary model excluding race | 2-days moving average | 223788 | 499 | 1.10 | 1.07 | 1.12 |
| Circulatory Hospital Admissions | Primary model excluding race | 3-days moving average | 223675 | 612 | 1.10 | 1.07 | 1.13 |
| Circulatory Hospital Admissions | Primary model excluding race | 4-days moving average | 223726 | 561 | 1.11 | 1.08 | 1.14 |
| Circulatory Hospital Admissions | Primary model excluding race | 5-days moving average | 223642 | 645 | 1.13 | 1.10 | 1.16 |
| Circulatory Hospital Admissions | Primary model excluding state and lat/long | 1-day moving average | 223733 | 554 | 1.10 | 1.08 | 1.12 |
| Circulatory Hospital Admissions | Primary model excluding state and lat/long | 2-days moving average | 223788 | 499 | 1.10 | 1.07 | 1.13 |
| Circulatory Hospital Admissions | Primary model excluding state and lat/long | 3-days moving average | 223675 | 612 | 1.10 | 1.07 | 1.13 |
| Circulatory Hospital Admissions | Primary model excluding state and lat/long | 4-days moving average | 223726 | 561 | 1.11 | 1.08 | 1.14 |
| Circulatory Hospital Admissions | Primary model excluding state and lat/long | 5-days moving average | 223642 | 645 | 1.13 | 1.10 | 1.16 |
| Respiratory Hospital Admissions | Primary analysis | 1-day moving average | 271665 | 662 | 1.11 | 1.09 | 1.13 |
| Respiratory Hospital Admissions | Primary analysis | 2-days moving average | 271683 | 644 | 1.12 | 1.09 | 1.15 |
| Respiratory Hospital Admissions | Primary analysis | 3-days moving average | 271561 | 766 | 1.17 | 1.14 | 1.19 |
| Respiratory Hospital Admissions | Primary analysis | 4-days moving average | 271877 | 450 | 1.18 | 1.18 | 1.18 |
| Respiratory Hospital Admissions | Primary analysis | 5-days moving average | 271878 | 449 | NA | NA | NA |
| Respiratory Hospital Admissions | Stratified by sex - Man | 1-day moving average | 140714 | 363 | 1.12 | 1.09 | 1.15 |
| Respiratory Hospital Admissions | Stratified by sex - Man | 2-days moving average | 140932 | 145 | 0.00 | 0.00 | 0.00 |
| Respiratory Hospital Admissions | Stratified by sex - Man | 3-days moving average | 140937 | 140 | 0.00 | 0.00 | 0.00 |
| Respiratory Hospital Admissions | Stratified by sex - Man | 4-days moving average | 140957 | 120 | 1.24 | 1.15 | 1.32 |
| Respiratory Hospital Admissions | Stratified by sex - Man | 5-days moving average | NA | NA | NA | NA | NA |
| Respiratory Hospital Admissions | Stratified by sex - Woman | 1-day moving average | 131130 | 120 | 0.00 | 0.00 | 0.00 |
| Respiratory Hospital Admissions | Stratified by sex - Woman | 2-days moving average | 131189 | 120 | 1.25 | 1.16 | 1.34 |
| Respiratory Hospital Admissions | Stratified by sex - Woman | 3-days moving average | NA | NA | NA | NA | NA |
| Respiratory Hospital Admissions | Stratified by sex - Woman | 4-days moving average | NA | NA | NA | NA | NA |
| Respiratory Hospital Admissions | Stratified by sex - Woman | 5-days moving average | 131176 | 111 | 1.23 | 1.12 | 1.35 |
| Respiratory Hospital Admissions | Stratified by age (0-5 years old) | 1-day moving average | 82222 | 196 | 1.13 | 1.08 | 1.19 |
| Respiratory Hospital Admissions | Stratified by age (0-5 years old) | 2-days moving average | 82222 | 196 | 1.13 | 1.08 | 1.19 |
| Respiratory Hospital Admissions | Stratified by age (0-5 years old) | 3-days moving average | 82187 | 231 | 1.19 | 1.14 | 1.24 |
| Respiratory Hospital Admissions | Stratified by age (0-5 years old) | 4-days moving average | 82203 | 215 | 1.24 | 1.19 | 1.30 |
| Respiratory Hospital Admissions | Stratified by age (0-5 years old) | 5-days moving average | 82190 | 228 | 1.18 | 1.13 | 1.24 |
| Respiratory Hospital Admissions | Stratified by age (35-64 years old) | 1-day moving average | 48215 | 123 | 1.15 | 1.10 | 1.21 |
| Respiratory Hospital Admissions | Stratified by age (35-64 years old) | 2-days moving average | 48223 | 115 | 1.13 | 1.06 | 1.20 |
| Respiratory Hospital Admissions | Stratified by age (35-64 years old) | 3-days moving average | 48196 | 142 | 1.18 | 1.12 | 1.25 |
| Respiratory Hospital Admissions | Stratified by age (35-64 years old) | 4-days moving average | 48189 | 149 | 1.12 | 1.05 | 1.19 |
| Respiratory Hospital Admissions | Stratified by age (35-64 years old) | 5-days moving average | 48218 | 120 | 1.12 | 1.03 | 1.20 |
| Respiratory Hospital Admissions | Stratified by age (> 64 years old) | 1-day moving average | 61066 | 129 | 1.16 | 1.09 | 1.23 |
| Respiratory Hospital Admissions | Stratified by age (> 64 years old) | 2-days moving average | 61065 | 130 | 1.15 | 1.09 | 1.22 |
| Respiratory Hospital Admissions | Stratified by age (> 64 years old) | 3-days moving average | 61049 | 146 | 1.22 | 1.15 | 1.29 |
| Respiratory Hospital Admissions | Stratified by age (> 64 years old) | 4-days moving average | 61051 | 144 | 1.21 | 1.13 | 1.28 |
| Respiratory Hospital Admissions | Stratified by age (> 64 years old) | 5-days moving average | 61043 | 152 | 1.16 | 1.09 | 1.24 |
| Respiratory Hospital Admissions | Primary model excluding the other pollutants (CO, NO2, and O3) | 1-day moving average | 271665 | 662 | 1.17 | 1.16 | 1.18 |
| Respiratory Hospital Admissions | Primary model excluding the other pollutants (CO, NO2, and O3) | 2-days moving average | 271683 | 644 | 1.20 | 1.19 | 1.21 |
| Respiratory Hospital Admissions | Primary model excluding the other pollutants (CO, NO2, and O3) | 3-days moving average | 271561 | 766 | 1.21 | 1.20 | 1.22 |
| Respiratory Hospital Admissions | Primary model excluding the other pollutants (CO, NO2, and O3) | 4-days moving average | 271585 | 742 | NA | NA | NA |
| Respiratory Hospital Admissions | Primary model excluding the other pollutants (CO, NO2, and O3) | 5-days moving average | 271609 | 718 | NA | NA | NA |
| Respiratory Hospital Admissions | Primary model excluding race | 1-day moving average | 271665 | 662 | 1.11 | 1.09 | 1.13 |
| Respiratory Hospital Admissions | Primary model excluding race | 2-days moving average | 271683 | 644 | 1.12 | 1.10 | 1.15 |
| Respiratory Hospital Admissions | Primary model excluding race | 3-days moving average | 271561 | 766 | 1.17 | 1.14 | 1.19 |
| Respiratory Hospital Admissions | Primary model excluding race | 4-days moving average | NA | NA | NA | NA | NA |
| Respiratory Hospital Admissions | Primary model excluding race | 5-days moving average | NA | NA | NA | NA | NA |
| Respiratory Hospital Admissions | Primary model excluding state and lat/long | 1-day moving average | 271665 | 662 | 1.11 | 1.09 | 1.13 |
| Respiratory Hospital Admissions | Primary model excluding state and lat/long | 2-days moving average | 271683 | 644 | 1.12 | 1.09 | 1.15 |
| Respiratory Hospital Admissions | Primary model excluding state and lat/long | 3-days moving average | 271561 | 766 | 1.17 | 1.14 | 1.19 |
| Respiratory Hospital Admissions | Primary model excluding state and lat/long | 4-days moving average | NA | NA | NA | NA | NA |
| Respiratory Hospital Admissions | Primary model excluding state and lat/long | 5-days moving average | 271609 | 718 | NA | NA | NA |

Table 3 (Appendix 2): Odds ratio and 95%CI for the North region. Results are stratified by health outcome, model (primary and sensitivity analyses), and moving average (1-5). Note: “NA” means that there were very large groups of ties (a large number of events out of a large number of subjects). The algorithm in the Survival package may refuse to undertake the task (the computation is infeasible) due to overflow for the subscripts.

| Outcome | Model | Moving Average | Controls(n) | Cases(n) | Odds Ratio | Lower 95%CI | Upper 95%CI |
| --- | --- | --- | --- | --- | --- | --- | --- |
| Cardiorespiratory Hospital Admissions | Primary analysis | 1-day moving average | 137300 | 182 | 1.14 | 1.10 | 1.17 |
| Cardiorespiratory Hospital Admissions | Primary analysis | 2-days moving average | 137318 | 164 | 1.14 | 1.11 | 1.17 |
| Cardiorespiratory Hospital Admissions | Primary analysis | 3-days moving average | 137332 | 150 | 1.15 | 1.11 | 1.19 |
| Cardiorespiratory Hospital Admissions | Primary analysis | 4-days moving average | 137308 | 174 | 1.17 | 1.12 | 1.22 |
| Cardiorespiratory Hospital Admissions | Primary analysis | 5-days moving average | 137273 | 209 | 1.36 | 1.29 | 1.42 |
| Cardiorespiratory Hospital Admissions | Stratified by sex - Man | 1-day moving average | 72301 | 94 | 1.14 | 1.08 | 1.20 |
| Cardiorespiratory Hospital Admissions | Stratified by sex - Man | 2-days moving average | 72311 | 84 | 1.12 | 1.08 | 1.17 |
| Cardiorespiratory Hospital Admissions | Stratified by sex - Man | 3-days moving average | 72316 | 79 | 1.11 | 1.06 | 1.16 |
| Cardiorespiratory Hospital Admissions | Stratified by sex - Man | 4-days moving average | 72311 | 84 | 1.12 | 1.06 | 1.19 |
| Cardiorespiratory Hospital Admissions | Stratified by sex - Man | 5-days moving average | 72297 | 98 | 1.25 | 1.17 | 1.33 |
| Cardiorespiratory Hospital Admissions | Stratified by sex - Woman | 1-day moving average | 64990 | 97 | 1.15 | 1.10 | 1.21 |
| Cardiorespiratory Hospital Admissions | Stratified by sex - Woman | 2-days moving average | 65010 | 77 | 1.21 | 1.15 | 1.27 |
| Cardiorespiratory Hospital Admissions | Stratified by sex - Woman | 3-days moving average | 65012 | 75 | 1.21 | 1.14 | 1.27 |
| Cardiorespiratory Hospital Admissions | Stratified by sex - Woman | 4-days moving average | 65000 | 87 | 1.30 | 1.20 | 1.41 |
| Cardiorespiratory Hospital Admissions | Stratified by sex - Woman | 5-days moving average | 64984 | 103 | 1.43 | 1.32 | 1.54 |
| Cardiorespiratory Hospital Admissions | Stratified by age (0-5 years old) | 1-day moving average | 32365 | 36 | 1.27 | 1.10 | 1.44 |
| Cardiorespiratory Hospital Admissions | Stratified by age (0-5 years old) | 2-days moving average | 32361 | 40 | 1.24 | 1.12 | 1.36 |
| Cardiorespiratory Hospital Admissions | Stratified by age (0-5 years old) | 3-days moving average | 32360 | 41 | 1.13 | 1.04 | 1.21 |
| Cardiorespiratory Hospital Admissions | Stratified by age (0-5 years old) | 4-days moving average | 32340 | 61 | 1.21 | 1.08 | 1.35 |
| Cardiorespiratory Hospital Admissions | Stratified by age (0-5 years old) | 5-days moving average | 32339 | 62 | NA | NA | NA |
| Cardiorespiratory Hospital Admissions | Stratified by age (35-64 years old) | 1-day moving average | 35506 | 46 | 1.40 | 1.17 | 1.63 |
| Cardiorespiratory Hospital Admissions | Stratified by age (35-64 years old) | 2-days moving average | 35516 | 36 | 1.27 | 1.17 | 1.38 |
| Cardiorespiratory Hospital Admissions | Stratified by age (35-64 years old) | 3-days moving average | 35523 | 29 | 1.19 | 1.09 | 1.30 |
| Cardiorespiratory Hospital Admissions | Stratified by age (35-64 years old) | 4-days moving average | 35521 | 31 | 1.28 | 1.11 | 1.44 |
| Cardiorespiratory Hospital Admissions | Stratified by age (35-64 years old) | 5-days moving average | 35515 | 37 | 1.34 | 1.19 | 1.48 |
| Cardiorespiratory Hospital Admissions | Stratified by age (> 64 years old) | 1-day moving average | 34714 | 51 | 1.15 | 1.07 | 1.22 |
| Cardiorespiratory Hospital Admissions | Stratified by age (> 64 years old) | 2-days moving average | 34720 | 45 | 1.43 | 1.23 | 1.62 |
| Cardiorespiratory Hospital Admissions | Stratified by age (> 64 years old) | 3-days moving average | 34722 | 43 | 1.20 | 1.03 | 1.37 |
| Cardiorespiratory Hospital Admissions | Stratified by age (> 64 years old) | 4-days moving average | 34719 | 46 | 1.19 | 1.07 | 1.30 |
| Cardiorespiratory Hospital Admissions | Stratified by age (> 64 years old) | 5-days moving average | NA | NA | NA | NA | NA |
| Cardiorespiratory Hospital Admissions | Primary model excluding the other pollutants (CO, NO2, and O3) | 1-day moving average | 137300 | 182 | 1.22 | 1.20 | 1.24 |
| Cardiorespiratory Hospital Admissions | Primary model excluding the other pollutants (CO, NO2, and O3) | 2-days moving average | 137318 | 164 | 1.17 | 1.15 | 1.19 |
| Cardiorespiratory Hospital Admissions | Primary model excluding the other pollutants (CO, NO2, and O3) | 3-days moving average | 137332 | 150 | 1.21 | 1.19 | 1.23 |
| Cardiorespiratory Hospital Admissions | Primary model excluding the other pollutants (CO, NO2, and O3) | 4-days moving average | 137308 | 174 | 1.27 | 1.24 | 1.30 |
| Cardiorespiratory Hospital Admissions | Primary model excluding the other pollutants (CO, NO2, and O3) | 5-days moving average | 137273 | 209 | 1.39 | 1.35 | 1.43 |
| Cardiorespiratory Hospital Admissions | Primary model excluding race | 1-day moving average | 137300 | 182 | 1.14 | 1.10 | 1.17 |
| Cardiorespiratory Hospital Admissions | Primary model excluding race | 2-days moving average | 137318 | 164 | 1.14 | 1.10 | 1.17 |
| Cardiorespiratory Hospital Admissions | Primary model excluding race | 3-days moving average | 137332 | 150 | 1.15 | 1.11 | 1.19 |
| Cardiorespiratory Hospital Admissions | Primary model excluding race | 4-days moving average | 137308 | 174 | 1.17 | 1.12 | 1.22 |
| Cardiorespiratory Hospital Admissions | Primary model excluding race | 5-days moving average | 137273 | 209 | 1.36 | 1.30 | 1.42 |
| Cardiorespiratory Hospital Admissions | Primary model excluding state and lat/long | 1-day moving average | 137300 | 182 | 1.14 | 1.10 | 1.17 |
| Cardiorespiratory Hospital Admissions | Primary model excluding state and lat/long | 2-days moving average | 137318 | 164 | 1.14 | 1.10 | 1.17 |
| Cardiorespiratory Hospital Admissions | Primary model excluding state and lat/long | 3-days moving average | 137332 | 150 | 1.15 | 1.12 | 1.19 |
| Cardiorespiratory Hospital Admissions | Primary model excluding state and lat/long | 4-days moving average | 137308 | 174 | 1.17 | 1.12 | 1.22 |
| Cardiorespiratory Hospital Admissions | Primary model excluding state and lat/long | 5-days moving average | 137273 | 209 | 1.31 | 1.25 | 1.36 |
| Circulatory Hospital Admissions | Primary analysis | 1-day moving average | 47441 | 64 | 1.18 | 1.08 | 1.28 |
| Circulatory Hospital Admissions | Primary analysis | 2-days moving average | 47447 | 58 | 1.23 | 1.14 | 1.31 |
| Circulatory Hospital Admissions | Primary analysis | 3-days moving average | 47456 | 49 | 1.24 | 1.12 | 1.36 |
| Circulatory Hospital Admissions | Primary analysis | 4-days moving average | 47461 | 44 | 1.20 | 1.07 | 1.33 |
| Circulatory Hospital Admissions | Primary analysis | 5-days moving average | 47465 | 40 | 1.28 | 1.15 | 1.40 |
| Circulatory Hospital Admissions | Stratified by sex - Man | 1-day moving average | 24766 | 25 | 1.81 | 0.54 | 3.08 |
| Circulatory Hospital Admissions | Stratified by sex - Man | 2-days moving average | 24765 | 26 | 1.30 | 1.11 | 1.50 |
| Circulatory Hospital Admissions | Stratified by sex - Man | 2-days moving average | 24766 | 25 | NA | NA | NA |
| Circulatory Hospital Admissions | Stratified by sex - Man | 4-days moving average | 24769 | 22 | 1.25 | 0.99 | 1.50 |
| Circulatory Hospital Admissions | Stratified by sex - Man | 5-days moving average | 24768 | 23 | 1.74 | 1.08 | 2.40 |
| Circulatory Hospital Admissions | Stratified by sex - Woman | 1-day moving average | 22677 | 37 | 1.34 | 1.13 | 1.55 |
| Circulatory Hospital Admissions | Stratified by sex - Woman | 2-days moving average | 22682 | 32 | 1.59 | 1.22 | 1.96 |
| Circulatory Hospital Admissions | Stratified by sex - Woman | 3-days moving average | NA | NA | NA | NA | NA |
| Circulatory Hospital Admissions | Stratified by sex - Woman | 4-days moving average | 22692 | 22 | NA | NA | NA |
| Circulatory Hospital Admissions | Stratified by sex - Woman | 5-days moving average | 22693 | 21 | NA | NA | NA |
| Circulatory Hospital Admissions | Stratified by age (0-5 years old) | 1-day moving average | 575 | NA | NA | NA | NA |
| Circulatory Hospital Admissions | Stratified by age (0-5 years old) | 2-days moving average | 574 | 1 | NA | NA | NA |
| Circulatory Hospital Admissions | Stratified by age (0-5 years old) | 3-days moving average | 575 | NA | NA | NA | NA |
| Circulatory Hospital Admissions | Stratified by age (0-5 years old) | 4-days moving average | 575 | NA | NA | NA | NA |
| Circulatory Hospital Admissions | Stratified by age (0-5 years old) | 5-days moving average | 575 | NA | NA | NA | NA |
| Circulatory Hospital Admissions | Stratified by age (35-64 years old) | 1-day moving average | NA | NA | NA | NA | NA |
| Circulatory Hospital Admissions | Stratified by age (35-64 years old) | 2-days moving average | NA | NA | NA | NA | NA |
| Circulatory Hospital Admissions | Stratified by age (35-64 years old) | 3-days moving average | 21137 | 19 | 2.39 | 1.36 | 3.42 |
| Circulatory Hospital Admissions | Stratified by age (35-64 years old) | 4-days moving average | NA | NA | NA | NA | NA |
| Circulatory Hospital Admissions | Stratified by age (35-64 years old) | 5-days moving average | NA | NA | NA | NA | NA |
| Circulatory Hospital Admissions | Stratified by age (> 64 years old) | 1-day moving average | 20356 | 27 | 25.20 | 25.20 | 25.20 |
| Circulatory Hospital Admissions | Stratified by age (> 64 years old) | 2-days moving average | 20356 | 27 | 31.50 | 31.50 | 31.50 |
| Circulatory Hospital Admissions | Stratified by age (> 64 years old) | 3-days moving average | NA | NA | NA | NA | NA |
| Circulatory Hospital Admissions | Stratified by age (> 64 years old) | 4-days moving average | 20357 | 26 | 0.81 | 0.81 | 0.81 |
| Circulatory Hospital Admissions | Stratified by age (> 64 years old) | 5-days moving average | NA | NA | NA | NA | NA |
| Circulatory Hospital Admissions | Primary model excluding the other pollutants (CO, NO2, and O3) | 1-day moving average | 47441 | 64 | 1.34 | 1.26 | 1.42 |
| Circulatory Hospital Admissions | Primary model excluding the other pollutants (CO, NO2, and O3) | 2-days moving average | 47447 | 58 | 1.24 | 1.19 | 1.29 |
| Circulatory Hospital Admissions | Primary model excluding the other pollutants (CO, NO2, and O3) | 3-days moving average | 47456 | 49 | 1.30 | 1.22 | 1.39 |
| Circulatory Hospital Admissions | Primary model excluding the other pollutants (CO, NO2, and O3) | 4-days moving average | 47461 | 44 | 1.33 | 1.23 | 1.42 |
| Circulatory Hospital Admissions | Primary model excluding the other pollutants (CO, NO2, and O3) | 5-days moving average | 47465 | 40 | 1.35 | 1.25 | 1.44 |
| Circulatory Hospital Admissions | Primary model excluding race | 1-day moving average | 47441 | 64 | 1.18 | 1.08 | 1.28 |
| Circulatory Hospital Admissions | Primary model excluding race | 2-days moving average | 47447 | 58 | 1.22 | 1.14 | 1.31 |
| Circulatory Hospital Admissions | Primary model excluding race | 3-days moving average | 47456 | 49 | 1.22 | 1.11 | 1.32 |
| Circulatory Hospital Admissions | Primary model excluding race | 4-days moving average | 47461 | 44 | 1.20 | 1.07 | 1.32 |
| Circulatory Hospital Admissions | Primary model excluding race | 5-days moving average | 47465 | 40 | 1.26 | 1.15 | 1.38 |
| Circulatory Hospital Admissions | Primary model excluding state and lat/long | 1-day moving average | 47441 | 64 | 1.18 | 1.09 | 1.28 |
| Circulatory Hospital Admissions | Primary model excluding state and lat/long | 2-days moving average | 47447 | 58 | 1.24 | 1.15 | 1.32 |
| Circulatory Hospital Admissions | Primary model excluding state and lat/long | 3-days moving average | 47456 | 49 | 1.23 | 1.12 | 1.34 |
| Circulatory Hospital Admissions | Primary model excluding state and lat/long | 4-days moving average | 47461 | 44 | 1.19 | 1.07 | 1.31 |
| Circulatory Hospital Admissions | Primary model excluding state and lat/long | 5-days moving average | 47465 | 40 | 1.25 | 1.14 | 1.36 |
| Respiratory Hospital Admissions | Primary analysis | 1-day moving average | 89856 | 121 | 1.14 | 1.10 | 1.19 |
| Respiratory Hospital Admissions | Primary analysis | 2-days moving average | 89869 | 108 | 1.12 | 1.08 | 1.16 |
| Respiratory Hospital Admissions | Primary analysis | 3-days moving average | 89869 | 108 | 1.14 | 1.09 | 1.19 |
| Respiratory Hospital Admissions | Primary analysis | 4-days moving average | 89846 | 131 | 1.19 | 1.12 | 1.25 |
| Respiratory Hospital Admissions | Primary analysis | 5-days moving average | 89809 | 168 | 1.39 | 1.31 | 1.47 |
| Respiratory Hospital Admissions | Stratified by sex - Man | 1-day moving average | 47539 | 65 | 1.11 | 1.05 | 1.18 |
| Respiratory Hospital Admissions | Stratified by sex - Man | 2-days moving average | 47545 | 59 | 1.19 | 1.11 | 1.27 |
| Respiratory Hospital Admissions | Stratified by sex - Man | 3-days moving average | 47549 | 55 | 1.11 | 1.05 | 1.16 |
| Respiratory Hospital Admissions | Stratified by sex - Man | 4-days moving average | 47541 | 63 | 1.21 | 1.10 | 1.32 |
| Respiratory Hospital Admissions | Stratified by sex - Man | 5-days moving average | 47521 | 83 | 1.47 | 1.32 | 1.62 |
| Respiratory Hospital Admissions | Stratified by sex - Woman | 1-day moving average | 42315 | 58 | 1.14 | 1.08 | 1.20 |
| Respiratory Hospital Admissions | Stratified by sex - Woman | 2-days moving average | 42329 | 44 | 1.21 | 1.12 | 1.31 |
| Respiratory Hospital Admissions | Stratified by sex - Woman | 3-days moving average | 42322 | 51 | 1.21 | 1.12 | 1.30 |
| Respiratory Hospital Admissions | Stratified by sex - Woman | 4-days moving average | 42305 | 68 | 1.25 | 1.13 | 1.36 |
| Respiratory Hospital Admissions | Stratified by sex - Woman | 5-days moving average | 42290 | 83 | 1.45 | 1.31 | 1.60 |
| Respiratory Hospital Admissions | Stratified by age (0-5 years old) | 1-day moving average | 31792 | 34 | 1.17 | 1.05 | 1.28 |
| Respiratory Hospital Admissions | Stratified by age (0-5 years old) | 2-days moving average | 31788 | 38 | 1.21 | 1.08 | 1.34 |
| Respiratory Hospital Admissions | Stratified by age (0-5 years old) | 3-days moving average | 31785 | 41 | 1.13 | 1.04 | 1.21 |
| Respiratory Hospital Admissions | Stratified by age (0-5 years old) | 4-days moving average | 31765 | 61 | 1.21 | 1.08 | 1.35 |
| Respiratory Hospital Admissions | Stratified by age (0-5 years old) | 5-days moving average | 31764 | 62 | NA | NA | NA |
| Respiratory Hospital Admissions | Stratified by age (35-64 years old) | 1-day moving average | NA | NA | NA | NA | NA |
| Respiratory Hospital Admissions | Stratified by age (35-64 years old) | 2-days moving average | NA | NA | NA | NA | NA |
| Respiratory Hospital Admissions | Stratified by age (35-64 years old) | 3-days moving average | 14383 | 13 | NA | NA | NA |
| Respiratory Hospital Admissions | Stratified by age (35-64 years old) | 4-days moving average | 14383 | 13 | NA | NA | NA |
| Respiratory Hospital Admissions | Stratified by age (35-64 years old) | 5-days moving average | NA | NA | NA | NA | NA |
| Respiratory Hospital Admissions | Stratified by age (> 64 years old) | 1-day moving average | 14357 | 25 | 1.12 | 1.01 | 1.22 |
| Respiratory Hospital Admissions | Stratified by age (> 64 years old) | 2-days moving average | 14363 | 19 | 2.41 | 0.77 | 4.04 |
| Respiratory Hospital Admissions | Stratified by age (> 64 years old) | 3-days moving average | 14367 | 15 | 1.04 | 0.88 | 1.19 |
| Respiratory Hospital Admissions | Stratified by age (> 64 years old) | 4-days moving average | 14364 | 18 | 1.19 | 1.03 | 1.36 |
| Respiratory Hospital Admissions | Stratified by age (> 64 years old) | 5-days moving average | 14359 | 23 | 8.65 | 3.54 | 13.75 |
| Respiratory Hospital Admissions | Primary model excluding the other pollutants (CO, NO2, and O3) | 1-day moving average | 89856 | 121 | 1.20 | 1.18 | 1.23 |
| Respiratory Hospital Admissions | Primary model excluding the other pollutants (CO, NO2, and O3) | 2-days moving average | 89869 | 108 | 1.18 | 1.15 | 1.20 |
| Respiratory Hospital Admissions | Primary model excluding the other pollutants (CO, NO2, and O3) | 3-days moving average | 89869 | 108 | 1.20 | 1.17 | 1.23 |
| Respiratory Hospital Admissions | Primary model excluding the other pollutants (CO, NO2, and O3) | 4-days moving average | 89846 | 131 | 1.27 | 1.24 | 1.31 |
| Respiratory Hospital Admissions | Primary model excluding the other pollutants (CO, NO2, and O3) | 5-days moving average | 89809 | 168 | 1.40 | 1.35 | 1.45 |
| Respiratory Hospital Admissions | Primary model excluding race | 1-day moving average | 89856 | 121 | 1.14 | 1.10 | 1.18 |
| Respiratory Hospital Admissions | Primary model excluding race | 2-days moving average | 89869 | 108 | 1.12 | 1.08 | 1.17 |
| Respiratory Hospital Admissions | Primary model excluding race | 3-days moving average | 89869 | 108 | 1.14 | 1.09 | 1.19 |
| Respiratory Hospital Admissions | Primary model excluding race | 4-days moving average | 89846 | 131 | 1.19 | 1.12 | 1.26 |
| Respiratory Hospital Admissions | Primary model excluding race | 5-days moving average | 89809 | 168 | 1.39 | 1.31 | 1.48 |
| Respiratory Hospital Admissions | Primary model excluding state and lat/long | 1-day moving average | 89856 | 121 | 1.14 | 1.10 | 1.18 |
| Respiratory Hospital Admissions | Primary model excluding state and lat/long | 2-days moving average | 89869 | 108 | 1.11 | 1.07 | 1.16 |
| Respiratory Hospital Admissions | Primary model excluding state and lat/long | 3-days moving average | 89869 | 108 | 1.14 | 1.09 | 1.19 |
| Respiratory Hospital Admissions | Primary model excluding state and lat/long | 4-days moving average | 89846 | 131 | 1.19 | 1.12 | 1.26 |
| Respiratory Hospital Admissions | Primary model excluding state and lat/long | 5-days moving average | 89809 | 168 | 1.34 | 1.27 | 1.42 |

Table 4 (Appendix 2): Odds ratio and 95%CI for the South East region. Results are stratified by health outcome, model (primary and sensitivity analyses), and moving average (1-5). Note: “NA” means that there were very large groups of ties (a large number of events out of a large number of subjects). The algorithm in the Survival package may refuse to undertake the task (the computation is infeasible) due to overflow for the subscripts.

| Outcome | Model | Moving Average | Controls(n) | Cases(n) | Odds Ratio | Lower 95%CI | Upper 95%CI |
| --- | --- | --- | --- | --- | --- | --- | --- |
| Cardiorespiratory Hospital Admissions | Primary analysis | 1-day moving average | 802087 | 263 | 1.09 | 1.08 | 1.11 |
| Cardiorespiratory Hospital Admissions | Primary analysis | 2-days moving average | 801752 | 598 | 1.09 | 1.08 | 1.10 |
| Cardiorespiratory Hospital Admissions | Primary analysis | 3-days moving average | 801771 | 579 | 1.10 | 1.08 | 1.11 |
| Cardiorespiratory Hospital Admissions | Primary analysis | 4-days moving average | 801504 | 846 | NA | NA | NA |
| Cardiorespiratory Hospital Admissions | Primary analysis | 5-days moving average | NA | NA | NA | NA | NA |
| Cardiorespiratory Hospital Admissions | Stratified by sex - Man | 1-day moving average | 418957 | 139 | 1.09 | 1.07 | 1.11 |
| Cardiorespiratory Hospital Admissions | Stratified by sex - Man | 2-days moving average | 418801 | 295 | 1.09 | 1.07 | 1.10 |
| Cardiorespiratory Hospital Admissions | Stratified by sex - Man | 3-days moving average | 418803 | 293 | 1.11 | 1.09 | 1.14 |
| Cardiorespiratory Hospital Admissions | Stratified by sex - Man | 4-days moving average | 418665 | 431 | 1.11 | 1.09 | 1.13 |
| Cardiorespiratory Hospital Admissions | Stratified by sex - Man | 5-days moving average | 418605 | 491 | 1.13 | 1.11 | 1.14 |
| Cardiorespiratory Hospital Admissions | Stratified by sex - Woman | 1-day moving average | 383125 | 129 | 1.15 | 1.11 | 1.18 |
| Cardiorespiratory Hospital Admissions | Stratified by sex - Woman | 2-days moving average | 382955 | 299 | 1.10 | 1.08 | 1.12 |
| Cardiorespiratory Hospital Admissions | Stratified by sex - Woman | 3-days moving average | 382971 | 283 | 1.08 | 1.06 | 1.11 |
| Cardiorespiratory Hospital Admissions | Stratified by sex - Woman | 4-days moving average | 382826 | 428 | NA | NA | NA |
| Cardiorespiratory Hospital Admissions | Stratified by sex - Woman | 5-days moving average | 382774 | 480 | 1.09 | 1.07 | 1.12 |
| Cardiorespiratory Hospital Admissions | Stratified by age (0-5 years old) | 1-day moving average | NA | NA | NA | NA | NA |
| Cardiorespiratory Hospital Admissions | Stratified by age (0-5 years old) | 2-days moving average | 86870 | 56 | 1.09 | 1.04 | 1.14 |
| Cardiorespiratory Hospital Admissions | Stratified by age (0-5 years old) | 3-days moving average | 86840 | 86 | 1.08 | 1.04 | 1.13 |
| Cardiorespiratory Hospital Admissions | Stratified by age (0-5 years old) | 4-days moving average | 86819 | 107 | 1.18 | 1.13 | 1.24 |
| Cardiorespiratory Hospital Admissions | Stratified by age (0-5 years old) | 5-days moving average | 86800 | 126 | 1.20 | 1.12 | 1.27 |
| Cardiorespiratory Hospital Admissions | Stratified by age (35-64 years old) | 1-day moving average | 293790 | 102 | 1.10 | 1.07 | 1.13 |
| Cardiorespiratory Hospital Admissions | Stratified by age (35-64 years old) | 2-days moving average | 293665 | 227 | 1.08 | 1.05 | 1.10 |
| Cardiorespiratory Hospital Admissions | Stratified by age (35-64 years old) | 3-days moving average | 293687 | 205 | 1.08 | 1.05 | 1.11 |
| Cardiorespiratory Hospital Admissions | Stratified by age (35-64 years old) | 4-days moving average | 293586 | 306 | 1.10 | 1.08 | 1.13 |
| Cardiorespiratory Hospital Admissions | Stratified by age (35-64 years old) | 5-days moving average | 293562 | 330 | 1.11 | 1.09 | 1.13 |
| Cardiorespiratory Hospital Admissions | Stratified by age (> 64 years old) | 1-day moving average | 308949 | 121 | 1.13 | 1.10 | 1.17 |
| Cardiorespiratory Hospital Admissions | Stratified by age (> 64 years old) | 2-days moving average | 308822 | 248 | 1.11 | 1.09 | 1.13 |
| Cardiorespiratory Hospital Admissions | Stratified by age (> 64 years old) | 3-days moving average | 308832 | 238 | 1.13 | 1.10 | 1.15 |
| Cardiorespiratory Hospital Admissions | Stratified by age (> 64 years old) | 4-days moving average | 308733 | 337 | 1.13 | 1.10 | 1.15 |
| Cardiorespiratory Hospital Admissions | Stratified by age (> 64 years old) | 5-days moving average | 308682 | 388 | 1.11 | 1.09 | 1.13 |
| Cardiorespiratory Hospital Admissions | Primary model excluding the other pollutants (CO, NO2, and O3) | 1-day moving average | 802087 | 263 | 1.17 | 1.16 | 1.18 |
| Cardiorespiratory Hospital Admissions | Primary model excluding the other pollutants (CO, NO2, and O3) | 2-days moving average | 801752 | 598 | 1.18 | 1.17 | 1.18 |
| Cardiorespiratory Hospital Admissions | Primary model excluding the other pollutants (CO, NO2, and O3) | 3-days moving average | 801771 | 579 | 1.18 | 1.17 | 1.19 |
| Cardiorespiratory Hospital Admissions | Primary model excluding the other pollutants (CO, NO2, and O3) | 4-days moving average | 801504 | 846 | NA | NA | NA |
| Cardiorespiratory Hospital Admissions | Primary model excluding the other pollutants (CO, NO2, and O3) | 5-days moving average | 801404 | 946 | NA | NA | NA |
| Cardiorespiratory Hospital Admissions | Primary model excluding race | 1-day moving average | 802087 | 263 | 1.09 | 1.08 | 1.11 |
| Cardiorespiratory Hospital Admissions | Primary model excluding race | 2-days moving average | 801752 | 598 | 1.09 | 1.08 | 1.10 |
| Cardiorespiratory Hospital Admissions | Primary model excluding race | 3-days moving average | 801771 | 579 | 1.10 | 1.08 | 1.11 |
| Cardiorespiratory Hospital Admissions | Primary model excluding race | 4-days moving average | 801504 | 846 | NA | NA | NA |
| Cardiorespiratory Hospital Admissions | Primary model excluding race | 5-days moving average | NA | NA | NA | NA | NA |
| Cardiorespiratory Hospital Admissions | Primary model excluding state and lat/long | 1-day moving average | 802087 | 263 | 1.09 | 1.08 | 1.11 |
| Cardiorespiratory Hospital Admissions | Primary model excluding state and lat/long | 2-days moving average | 801752 | 598 | 1.09 | 1.08 | 1.10 |
| Cardiorespiratory Hospital Admissions | Primary model excluding state and lat/long | 3-days moving average | 801771 | 579 | 1.10 | 1.08 | 1.11 |
| Cardiorespiratory Hospital Admissions | Primary model excluding state and lat/long | 4-days moving average | 801504 | 846 | NA | NA | NA |
| Cardiorespiratory Hospital Admissions | Primary model excluding state and lat/long | 5-days moving average | NA | NA | NA | NA | NA |
| Circulatory Hospital Admissions | Primary analysis | 1-day moving average | 447740 | 165 | 1.14 | 1.11 | 1.16 |
| Circulatory Hospital Admissions | Primary analysis | 2-days moving average | 447564 | 341 | 1.10 | 1.08 | 1.12 |
| Circulatory Hospital Admissions | Primary analysis | 3-days moving average | 447582 | 323 | 1.10 | 1.08 | 1.13 |
| Circulatory Hospital Admissions | Primary analysis | 4-days moving average | 447420 | 485 | NA | NA | NA |
| Circulatory Hospital Admissions | Primary analysis | 5-days moving average | 447355 | 550 | 1.10 | 1.08 | 1.12 |
| Circulatory Hospital Admissions | Stratified by sex - Man | 1-day moving average | 228191 | 85 | 1.12 | 1.09 | 1.16 |
| Circulatory Hospital Admissions | Stratified by sex - Man | 2-days moving average | 228118 | 158 | 1.11 | 1.09 | 1.14 |
| Circulatory Hospital Admissions | Stratified by sex - Man | 3-days moving average | 228106 | 170 | 1.13 | 1.10 | 1.16 |
| Circulatory Hospital Admissions | Stratified by sex - Man | 4-days moving average | 228031 | 245 | 1.11 | 1.09 | 1.14 |
| Circulatory Hospital Admissions | Stratified by sex - Man | 5-days moving average | 227993 | 283 | 1.13 | 1.11 | 1.15 |
| Circulatory Hospital Admissions | Stratified by sex - Woman | 1-day moving average | 219544 | 85 | 1.14 | 1.10 | 1.18 |
| Circulatory Hospital Admissions | Stratified by sex - Woman | 2-days moving average | 219448 | 181 | 1.11 | 1.08 | 1.14 |
| Circulatory Hospital Admissions | Stratified by sex - Woman | 3-days moving average | 219475 | 154 | 1.06 | 1.03 | 1.09 |
| Circulatory Hospital Admissions | Stratified by sex - Woman | 4-days moving average | 219384 | 245 | 1.09 | 1.06 | 1.13 |
| Circulatory Hospital Admissions | Stratified by sex - Woman | 5-days moving average | 219356 | 273 | 1.07 | 1.04 | 1.10 |
| Circulatory Hospital Admissions | Stratified by age (0-5 years old) | 1-day moving average | NA | NA | NA | NA | NA |
| Circulatory Hospital Admissions | Stratified by age (0-5 years old) | 2-days moving average | NA | NA | NA | NA | NA |
| Circulatory Hospital Admissions | Stratified by age (0-5 years old) | 3-days moving average | NA | NA | NA | NA | NA |
| Circulatory Hospital Admissions | Stratified by age (0-5 years old) | 4-days moving average | NA | NA | NA | NA | NA |
| Circulatory Hospital Admissions | Stratified by age (0-5 years old) | 5-days moving average | NA | NA | NA | NA | NA |
| Circulatory Hospital Admissions | Stratified by age (35-64 years old) | 1-day moving average | 216662 | 74 | 1.14 | 1.10 | 1.19 |
| Circulatory Hospital Admissions | Stratified by age (35-64 years old) | 2-days moving average | 216576 | 160 | 1.11 | 1.08 | 1.14 |
| Circulatory Hospital Admissions | Stratified by age (35-64 years old) | 3-days moving average | 216597 | 139 | 1.11 | 1.07 | 1.15 |
| Circulatory Hospital Admissions | Stratified by age (35-64 years old) | 4-days moving average | 216519 | 217 | 1.10 | 1.07 | 1.13 |
| Circulatory Hospital Admissions | Stratified by age (35-64 years old) | 5-days moving average | 216501 | 235 | 1.12 | 1.09 | 1.15 |
| Circulatory Hospital Admissions | Stratified by age (> 64 years old) | 1-day moving average | 199901 | 84 | 1.17 | 1.13 | 1.21 |
| Circulatory Hospital Admissions | Stratified by age (> 64 years old) | 2-days moving average | 199823 | 162 | 1.11 | 1.08 | 1.13 |
| Circulatory Hospital Admissions | Stratified by age (> 64 years old) | 3-days moving average | 199825 | 160 | 1.14 | 1.11 | 1.17 |
| Circulatory Hospital Admissions | Stratified by age (> 64 years old) | 4-days moving average | 199749 | 236 | 1.13 | 1.10 | 1.15 |
| Circulatory Hospital Admissions | Stratified by age (> 64 years old) | 5-days moving average | 199711 | 274 | 1.11 | 1.08 | 1.14 |
| Circulatory Hospital Admissions | Primary model excluding the other pollutants (CO, NO2, and O3) | 1-day moving average | 447740 | 165 | 1.19 | 1.17 | 1.21 |
| Circulatory Hospital Admissions | Primary model excluding the other pollutants (CO, NO2, and O3) | 2-days moving average | 447564 | 341 | 1.19 | 1.18 | 1.20 |
| Circulatory Hospital Admissions | Primary model excluding the other pollutants (CO, NO2, and O3) | 3-days moving average | 447582 | 323 | 1.18 | 1.17 | 1.20 |
| Circulatory Hospital Admissions | Primary model excluding the other pollutants (CO, NO2, and O3) | 4-days moving average | 447420 | 485 | NA | NA | NA |
| Circulatory Hospital Admissions | Primary model excluding the other pollutants (CO, NO2, and O3) | 5-days moving average | 447355 | 550 | 1.20 | 1.19 | 1.21 |
| Circulatory Hospital Admissions | Primary model excluding race | 1-day moving average | 447740 | 165 | 1.13 | 1.11 | 1.16 |
| Circulatory Hospital Admissions | Primary model excluding race | 2-days moving average | 447564 | 341 | 1.10 | 1.08 | 1.12 |
| Circulatory Hospital Admissions | Primary model excluding race | 3-days moving average | 447582 | 323 | 1.10 | 1.08 | 1.13 |
| Circulatory Hospital Admissions | Primary model excluding race | 4-days moving average | 447420 | 485 | 1.10 | 1.08 | 1.12 |
| Circulatory Hospital Admissions | Primary model excluding race | 5-days moving average | 447355 | 550 | 1.10 | 1.08 | 1.12 |
| Circulatory Hospital Admissions | Primary model excluding state and lat/long | 1-day moving average | 447740 | 165 | 1.13 | 1.11 | 1.16 |
| Circulatory Hospital Admissions | Primary model excluding state and lat/long | 2-days moving average | 447564 | 341 | 1.10 | 1.08 | 1.12 |
| Circulatory Hospital Admissions | Primary model excluding state and lat/long | 3-days moving average | 447582 | 323 | 1.10 | 1.08 | 1.13 |
| Circulatory Hospital Admissions | Primary model excluding state and lat/long | 4-days moving average | 447420 | 485 | 1.10 | 1.08 | 1.12 |
| Circulatory Hospital Admissions | Primary model excluding state and lat/long | 5-days moving average | 447355 | 550 | 1.10 | 1.08 | 1.12 |
| Respiratory Hospital Admissions | Primary analysis | 1-day moving average | 354340 | 105 | 1.09 | 1.07 | 1.11 |
| Respiratory Hospital Admissions | Primary analysis | 2-days moving average | 354189 | 256 | 1.08 | 1.06 | 1.09 |
| Respiratory Hospital Admissions | Primary analysis | 3-days moving average | 354194 | 251 | 1.09 | 1.07 | 1.11 |
| Respiratory Hospital Admissions | Primary analysis | 4-days moving average | 354082 | 363 | 1.13 | 1.11 | 1.16 |
| Respiratory Hospital Admissions | Primary analysis | 5-days moving average | 354030 | 415 | 1.13 | 1.11 | 1.15 |
| Respiratory Hospital Admissions | Stratified by sex - Man | 1-day moving average | 190761 | 59 | 1.08 | 1.04 | 1.12 |
| Respiratory Hospital Admissions | Stratified by sex - Man | 2-days moving average | 190682 | 138 | 1.06 | 1.03 | 1.10 |
| Respiratory Hospital Admissions | Stratified by sex - Man | 3-days moving average | 190698 | 122 | 1.09 | 1.05 | 1.12 |
| Respiratory Hospital Admissions | Stratified by sex - Man | 4-days moving average | 190638 | 182 | 1.13 | 1.10 | 1.17 |
| Respiratory Hospital Admissions | Stratified by sex - Man | 5-days moving average | 190611 | 209 | 1.14 | 1.11 | 1.18 |
| Respiratory Hospital Admissions | Stratified by sex - Woman | 1-day moving average | 163579 | 46 | 1.15 | 1.10 | 1.20 |
| Respiratory Hospital Admissions | Stratified by sex - Woman | 2-days moving average | 163507 | 118 | 1.08 | 1.06 | 1.11 |
| Respiratory Hospital Admissions | Stratified by sex - Woman | 3-days moving average | 163496 | 129 | 1.10 | 1.07 | 1.13 |
| Respiratory Hospital Admissions | Stratified by sex - Woman | 4-days moving average | 163443 | 182 | 1.14 | 1.11 | 1.17 |
| Respiratory Hospital Admissions | Stratified by sex - Woman | 5-days moving average | 163420 | 205 | 1.13 | 1.10 | 1.17 |
| Respiratory Hospital Admissions | Stratified by age (0-5 years old) | 1-day moving average | NA | NA | NA | NA | NA |
| Respiratory Hospital Admissions | Stratified by age (0-5 years old) | 2-days moving average | 84380 | 57 | 1.09 | 1.04 | 1.13 |
| Respiratory Hospital Admissions | Stratified by age (0-5 years old) | 3-days moving average | 84354 | 83 | 1.11 | 1.06 | 1.16 |
| Respiratory Hospital Admissions | Stratified by age (0-5 years old) | 4-days moving average | 84333 | 104 | 1.19 | 1.13 | 1.25 |
| Respiratory Hospital Admissions | Stratified by age (0-5 years old) | 5-days moving average | 84315 | 122 | 1.24 | 1.16 | 1.32 |
| Respiratory Hospital Admissions | Stratified by age (35-64 years old) | 1-day moving average | 77128 | 28 | 1.10 | 1.05 | 1.16 |
| Respiratory Hospital Admissions | Stratified by age (35-64 years old) | 2-days moving average | 77088 | 68 | 1.02 | 0.97 | 1.08 |
| Respiratory Hospital Admissions | Stratified by age (35-64 years old) | 3-days moving average | 77088 | 68 | 1.03 | 0.98 | 1.09 |
| Respiratory Hospital Admissions | Stratified by age (35-64 years old) | 4-days moving average | 77069 | 87 | 1.14 | 1.08 | 1.21 |
| Respiratory Hospital Admissions | Stratified by age (35-64 years old) | 5-days moving average | 77061 | 95 | 1.08 | 1.02 | 1.13 |
| Respiratory Hospital Admissions | Stratified by age (> 64 years old) | 1-day moving average | 109037 | 48 | 1.09 | 1.05 | 1.13 |
| Respiratory Hospital Admissions | Stratified by age (> 64 years old) | 2-days moving average | 108996 | 89 | 1.10 | 1.06 | 1.13 |
| Respiratory Hospital Admissions | Stratified by age (> 64 years old) | 3-days moving average | 109013 | 72 | 1.14 | 1.10 | 1.19 |
| Respiratory Hospital Admissions | Stratified by age (> 64 years old) | 4-days moving average | 108981 | 104 | 1.17 | 1.13 | 1.22 |
| Respiratory Hospital Admissions | Stratified by age (> 64 years old) | 5-days moving average | 108967 | 118 | 1.16 | 1.12 | 1.19 |
| Respiratory Hospital Admissions | Primary model excluding the other pollutants (CO, NO2, and O3) | 1-day moving average | 354340 | 105 | 1.16 | 1.14 | 1.17 |
| Respiratory Hospital Admissions | Primary model excluding the other pollutants (CO, NO2, and O3) | 2-days moving average | 354189 | 256 | 1.16 | 1.15 | 1.17 |
| Respiratory Hospital Admissions | Primary model excluding the other pollutants (CO, NO2, and O3) | 3-days moving average | 354194 | 251 | 1.18 | 1.17 | 1.20 |
| Respiratory Hospital Admissions | Primary model excluding the other pollutants (CO, NO2, and O3) | 4-days moving average | 354082 | 363 | NA | NA | NA |
| Respiratory Hospital Admissions | Primary model excluding the other pollutants (CO, NO2, and O3) | 5-days moving average | 354030 | 415 | 1.24 | 1.22 | 1.25 |
| Respiratory Hospital Admissions | Primary model excluding race | 1-day moving average | 354340 | 105 | 1.08 | 1.06 | 1.10 |
| Respiratory Hospital Admissions | Primary model excluding race | 2-days moving average | 354189 | 256 | 1.08 | 1.06 | 1.09 |
| Respiratory Hospital Admissions | Primary model excluding race | 3-days moving average | 354194 | 251 | 1.09 | 1.07 | 1.11 |
| Respiratory Hospital Admissions | Primary model excluding race | 4-days moving average | 354082 | 363 | 1.13 | 1.11 | 1.16 |
| Respiratory Hospital Admissions | Primary model excluding race | 5-days moving average | 354030 | 415 | 1.13 | 1.11 | 1.15 |
| Respiratory Hospital Admissions | Primary model excluding state and lat/long | 1-day moving average | 354340 | 105 | 1.09 | 1.07 | 1.11 |
| Respiratory Hospital Admissions | Primary model excluding state and lat/long | 2-days moving average | 354189 | 256 | 1.08 | 1.06 | 1.09 |
| Respiratory Hospital Admissions | Primary model excluding state and lat/long | 3-days moving average | 354194 | 251 | 1.09 | 1.07 | 1.11 |
| Respiratory Hospital Admissions | Primary model excluding state and lat/long | 4-days moving average | 354082 | 363 | 1.13 | 1.11 | 1.16 |
| Respiratory Hospital Admissions | Primary model excluding state and lat/long | 5-days moving average | 354030 | 415 | 1.13 | 1.10 | 1.15 |

Table 5 (Appendix 2): Odds ratio and 95%CI for the South region. Results are stratified by health outcome, model (primary and sensitivity analyses), and moving average (1-5). Note: “NA” means that there were very large groups of ties (a large number of events out of a large number of subjects). The algorithm in the Survival package may refuse to undertake the task (the computation is infeasible) due to overflow for the subscripts.

| Outcome | Model | Moving Average | Controls(n) | Cases(n) | Odds Ratio | Lower 95%CI | Upper 95%CI |
| --- | --- | --- | --- | --- | --- | --- | --- |
| Cardiorespiratory Hospital Admissions | Primary analysis | 1-day moving average | 427520 | 238 | 1.22 | 1.18 | 1.26 |
| Cardiorespiratory Hospital Admissions | Primary analysis | 2-days moving average | 427529 | 229 | 1.22 | 1.18 | 1.26 |
| Cardiorespiratory Hospital Admissions | Primary analysis | 3-days moving average | 427511 | 247 | 1.20 | 1.16 | 1.23 |
| Cardiorespiratory Hospital Admissions | Primary analysis | 4-days moving average | 427467 | 291 | 1.19 | 1.16 | 1.23 |
| Cardiorespiratory Hospital Admissions | Primary analysis | 5-days moving average | 427436 | 322 | 1.20 | 1.17 | 1.24 |
| Cardiorespiratory Hospital Admissions | Stratified by sex - Man | 1-day moving average | 219706 | 121 | 1.20 | 1.15 | 1.26 |
| Cardiorespiratory Hospital Admissions | Stratified by sex - Man | 2-days moving average | 219705 | 122 | 1.18 | 1.12 | 1.24 |
| Cardiorespiratory Hospital Admissions | Stratified by sex - Man | 3-days moving average | 219696 | 131 | 1.19 | 1.14 | 1.24 |
| Cardiorespiratory Hospital Admissions | Stratified by sex - Man | 4-days moving average | 219678 | 149 | 1.19 | 1.14 | 1.24 |
| Cardiorespiratory Hospital Admissions | Stratified by sex - Man | 5-days moving average | 219652 | 175 | 1.20 | 1.15 | 1.25 |
| Cardiorespiratory Hospital Admissions | Stratified by sex - Woman | 1-day moving average | 207815 | 116 | 1.23 | 1.18 | 1.29 |
| Cardiorespiratory Hospital Admissions | Stratified by sex - Woman | 2-days moving average | 207824 | 107 | 1.26 | 1.20 | 1.32 |
| Cardiorespiratory Hospital Admissions | Stratified by sex - Woman | 3-days moving average | 207815 | 116 | 1.24 | 1.16 | 1.31 |
| Cardiorespiratory Hospital Admissions | Stratified by sex - Woman | 4-days moving average | 207789 | 142 | 1.22 | 1.16 | 1.28 |
| Cardiorespiratory Hospital Admissions | Stratified by sex - Woman | 5-days moving average | 207784 | 147 | 1.23 | 1.17 | 1.29 |
| Cardiorespiratory Hospital Admissions | Stratified by age (0-5 years old) | 1-day moving average | 42186 | 16 | 1.98 | 0.87 | 3.08 |
| Cardiorespiratory Hospital Admissions | Stratified by age (0-5 years old) | 2-days moving average | NA | NA | NA | NA | NA |
| Cardiorespiratory Hospital Admissions | Stratified by age (0-5 years old) | 3-days moving average | NA | NA | NA | NA | NA |
| Cardiorespiratory Hospital Admissions | Stratified by age (0-5 years old) | 4-days moving average | 42178 | 24 | 2.32 | 1.58 | 3.06 |
| Cardiorespiratory Hospital Admissions | Stratified by age (0-5 years old) | 5-days moving average | 42170 | 32 | 1.73 | 1.33 | 2.14 |
| Cardiorespiratory Hospital Admissions | Stratified by age (35-64 years old) | 1-day moving average | 152659 | 85 | 1.20 | 1.14 | 1.26 |
| Cardiorespiratory Hospital Admissions | Stratified by age (35-64 years old) | 2-days moving average | 152662 | 82 | 1.17 | 1.10 | 1.23 |
| Cardiorespiratory Hospital Admissions | Stratified by age (35-64 years old) | 3-days moving average | 152655 | 89 | 1.18 | 1.11 | 1.25 |
| Cardiorespiratory Hospital Admissions | Stratified by age (35-64 years old) | 4-days moving average | 152648 | 96 | 1.24 | 1.17 | 1.30 |
| Cardiorespiratory Hospital Admissions | Stratified by age (35-64 years old) | 5-days moving average | 152633 | 111 | 1.25 | 1.19 | 1.32 |
| Cardiorespiratory Hospital Admissions | Stratified by age (> 64 years old) | 1-day moving average | 174317 | 102 | 1.31 | 1.24 | 1.39 |
| Cardiorespiratory Hospital Admissions | Stratified by age (> 64 years old) | 2-days moving average | 174321 | 98 | 1.24 | 1.17 | 1.30 |
| Cardiorespiratory Hospital Admissions | Stratified by age (> 64 years old) | 3-days moving average | 174317 | 102 | 1.19 | 1.13 | 1.24 |
| Cardiorespiratory Hospital Admissions | Stratified by age (> 64 years old) | 4-days moving average | 174289 | 130 | 1.17 | 1.13 | 1.22 |
| Cardiorespiratory Hospital Admissions | Stratified by age (> 64 years old) | 5-days moving average | 174283 | 136 | 1.19 | 1.13 | 1.24 |
| Cardiorespiratory Hospital Admissions | Primary model excluding the other pollutants (CO, NO2, and O3) | 1-day moving average | 427520 | 238 | 1.28 | 1.26 | 1.31 |
| Cardiorespiratory Hospital Admissions | Primary model excluding the other pollutants (CO, NO2, and O3) | 2-days moving average | 427529 | 229 | 1.28 | 1.25 | 1.30 |
| Cardiorespiratory Hospital Admissions | Primary model excluding the other pollutants (CO, NO2, and O3) | 3-days moving average | 427511 | 247 | 1.30 | 1.27 | 1.32 |
| Cardiorespiratory Hospital Admissions | Primary model excluding the other pollutants (CO, NO2, and O3) | 4-days moving average | 427467 | 291 | 1.28 | 1.26 | 1.30 |
| Cardiorespiratory Hospital Admissions | Primary model excluding the other pollutants (CO, NO2, and O3) | 5-days moving average | 427436 | 322 | 1.29 | 1.27 | 1.31 |
| Cardiorespiratory Hospital Admissions | Primary model excluding race | 1-day moving average | 427520 | 238 | 1.22 | 1.18 | 1.25 |
| Cardiorespiratory Hospital Admissions | Primary model excluding race | 2-days moving average | 427529 | 229 | 1.21 | 1.17 | 1.26 |
| Cardiorespiratory Hospital Admissions | Primary model excluding race | 3-days moving average | 427511 | 247 | 1.19 | 1.16 | 1.23 |
| Cardiorespiratory Hospital Admissions | Primary model excluding race | 4-days moving average | 427467 | 291 | 1.19 | 1.16 | 1.23 |
| Cardiorespiratory Hospital Admissions | Primary model excluding race | 5-days moving average | 427436 | 322 | 1.20 | 1.17 | 1.24 |
| Cardiorespiratory Hospital Admissions | Primary model excluding state and lat/long | 1-day moving average | 427520 | 238 | 1.20 | 1.16 | 1.24 |
| Cardiorespiratory Hospital Admissions | Primary model excluding state and lat/long | 2-days moving average | 427529 | 229 | 1.21 | 1.17 | 1.25 |
| Cardiorespiratory Hospital Admissions | Primary model excluding state and lat/long | 3-days moving average | 427511 | 247 | 1.19 | 1.15 | 1.23 |
| Cardiorespiratory Hospital Admissions | Primary model excluding state and lat/long | 4-days moving average | 427467 | 291 | 1.19 | 1.15 | 1.22 |
| Cardiorespiratory Hospital Admissions | Primary model excluding state and lat/long | 5-days moving average | 427436 | 322 | 1.20 | 1.17 | 1.24 |
| Circulatory Hospital Admissions | Primary analysis | 1-day moving average | 214304 | 124 | 1.22 | 1.17 | 1.26 |
| Circulatory Hospital Admissions | Primary analysis | 2-days moving average | 214309 | 119 | 1.21 | 1.16 | 1.27 |
| Circulatory Hospital Admissions | Primary analysis | 3-days moving average | 214306 | 122 | 1.19 | 1.13 | 1.25 |
| Circulatory Hospital Admissions | Primary analysis | 4-days moving average | 214285 | 143 | 1.20 | 1.15 | 1.25 |
| Circulatory Hospital Admissions | Primary analysis | 5-days moving average | 214263 | 165 | 1.22 | 1.17 | 1.27 |
| Circulatory Hospital Admissions | Stratified by sex - Man | 1-day moving average | 107188 | 66 | 1.22 | 1.14 | 1.31 |
| Circulatory Hospital Admissions | Stratified by sex - Man | 2-days moving average | 107186 | 68 | 1.13 | 1.05 | 1.21 |
| Circulatory Hospital Admissions | Stratified by sex - Man | 3-days moving average | 107190 | 64 | 1.17 | 1.08 | 1.25 |
| Circulatory Hospital Admissions | Stratified by sex - Man | 4-days moving average | 107179 | 75 | 1.18 | 1.11 | 1.25 |
| Circulatory Hospital Admissions | Stratified by sex - Man | 5-days moving average | 107168 | 86 | 1.25 | 1.18 | 1.33 |
| Circulatory Hospital Admissions | Stratified by sex - Woman | 1-day moving average | 107116 | 58 | 1.25 | 1.17 | 1.32 |
| Circulatory Hospital Admissions | Stratified by sex - Woman | 2-days moving average | 107123 | 51 | 1.33 | 1.24 | 1.43 |
| Circulatory Hospital Admissions | Stratified by sex - Woman | 3-days moving average | 107116 | 58 | 1.31 | 1.20 | 1.43 |
| Circulatory Hospital Admissions | Stratified by sex - Woman | 4-days moving average | 107106 | 68 | 1.30 | 1.20 | 1.39 |
| Circulatory Hospital Admissions | Stratified by sex - Woman | 5-days moving average | 107099 | 75 | 1.24 | 1.15 | 1.32 |
| Circulatory Hospital Admissions | Stratified by age (0-5 years old) | 1-day moving average | 1057 | NA | NA | NA | NA |
| Circulatory Hospital Admissions | Stratified by age (0-5 years old) | 2-days moving average | NA | NA | NA | NA | NA |
| Circulatory Hospital Admissions | Stratified by age (0-5 years old) | 3-days moving average | NA | NA | NA | NA | NA |
| Circulatory Hospital Admissions | Stratified by age (0-5 years old) | 4-days moving average | NA | NA | NA | NA | NA |
| Circulatory Hospital Admissions | Stratified by age (0-5 years old) | 5-days moving average | 1057 | NA | NA | NA | NA |
| Circulatory Hospital Admissions | Stratified by age (35-64 years old) | 1-day moving average | 100226 | 58 | 1.17 | 1.09 | 1.25 |
| Circulatory Hospital Admissions | Stratified by age (35-64 years old) | 2-days moving average | 100232 | 52 | 1.15 | 1.06 | 1.25 |
| Circulatory Hospital Admissions | Stratified by age (35-64 years old) | 3-days moving average | 100226 | 58 | 1.19 | 1.09 | 1.29 |
| Circulatory Hospital Admissions | Stratified by age (35-64 years old) | 4-days moving average | 100223 | 61 | 1.28 | 1.18 | 1.38 |
| Circulatory Hospital Admissions | Stratified by age (35-64 years old) | 5-days moving average | 100210 | 74 | 1.29 | 1.20 | 1.38 |
| Circulatory Hospital Admissions | Stratified by age (> 64 years old) | 1-day moving average | 101660 | 59 | 1.42 | 1.30 | 1.53 |
| Circulatory Hospital Admissions | Stratified by age (> 64 years old) | 2-days moving average | 101658 | 61 | 1.36 | 1.25 | 1.47 |
| Circulatory Hospital Admissions | Stratified by age (> 64 years old) | 3-days moving average | 101660 | 59 | 1.30 | 1.19 | 1.41 |
| Circulatory Hospital Admissions | Stratified by age (> 64 years old) | 4-days moving average | 101646 | 73 | 1.26 | 1.18 | 1.35 |
| Circulatory Hospital Admissions | Stratified by age (> 64 years old) | 5-days moving average | 101637 | 82 | 1.27 | 1.19 | 1.35 |
| Circulatory Hospital Admissions | Primary model excluding the other pollutants (CO, NO2, and O3) | 1-day moving average | 214304 | 124 | 1.26 | 1.23 | 1.30 |
| Circulatory Hospital Admissions | Primary model excluding the other pollutants (CO, NO2, and O3) | 2-days moving average | 214309 | 119 | 1.28 | 1.24 | 1.32 |
| Circulatory Hospital Admissions | Primary model excluding the other pollutants (CO, NO2, and O3) | 3-days moving average | 214306 | 122 | 1.30 | 1.26 | 1.34 |
| Circulatory Hospital Admissions | Primary model excluding the other pollutants (CO, NO2, and O3) | 4-days moving average | 214285 | 143 | 1.28 | 1.24 | 1.31 |
| Circulatory Hospital Admissions | Primary model excluding the other pollutants (CO, NO2, and O3) | 5-days moving average | 214263 | 165 | 1.30 | 1.27 | 1.33 |
| Circulatory Hospital Admissions | Primary model excluding race | 1-day moving average | 214304 | 124 | 1.21 | 1.16 | 1.25 |
| Circulatory Hospital Admissions | Primary model excluding race | 2-days moving average | 214309 | 119 | 1.21 | 1.15 | 1.27 |
| Circulatory Hospital Admissions | Primary model excluding race | 3-days moving average | 214306 | 122 | 1.19 | 1.13 | 1.25 |
| Circulatory Hospital Admissions | Primary model excluding race | 4-days moving average | 214285 | 143 | 1.20 | 1.15 | 1.25 |
| Circulatory Hospital Admissions | Primary model excluding race | 5-days moving average | 214263 | 165 | 1.22 | 1.17 | 1.27 |
| Circulatory Hospital Admissions | Primary model excluding state and lat/long | 1-day moving average | 214304 | 124 | 1.20 | 1.15 | 1.25 |
| Circulatory Hospital Admissions | Primary model excluding state and lat/long | 2-days moving average | 214309 | 119 | 1.20 | 1.14 | 1.26 |
| Circulatory Hospital Admissions | Primary model excluding state and lat/long | 3-days moving average | 214306 | 122 | 1.18 | 1.12 | 1.25 |
| Circulatory Hospital Admissions | Primary model excluding state and lat/long | 4-days moving average | 214285 | 143 | 1.19 | 1.14 | 1.25 |
| Circulatory Hospital Admissions | Primary model excluding state and lat/long | 5-days moving average | 214263 | 165 | 1.22 | 1.17 | 1.27 |
| Respiratory Hospital Admissions | Primary analysis | 1-day moving average | 213206 | 124 | 1.16 | 1.10 | 1.22 |
| Respiratory Hospital Admissions | Primary analysis | 2-days moving average | 213216 | 114 | 1.23 | 1.17 | 1.28 |
| Respiratory Hospital Admissions | Primary analysis | 3-days moving average | 213208 | 122 | 1.22 | 1.16 | 1.27 |
| Respiratory Hospital Admissions | Primary analysis | 4-days moving average | 213187 | 143 | 1.22 | 1.16 | 1.28 |
| Respiratory Hospital Admissions | Primary analysis | 5-days moving average | 213161 | 169 | 1.21 | 1.16 | 1.27 |
| Respiratory Hospital Admissions | Stratified by sex - Man | 1-day moving average | 112513 | 60 | 1.25 | 1.25 | 1.25 |
| Respiratory Hospital Admissions | Stratified by sex - Man | 2-days moving average | 112516 | 57 | 1.36 | 1.24 | 1.48 |
| Respiratory Hospital Admissions | Stratified by sex - Man | 3-days moving average | 112510 | 63 | 1.24 | 1.16 | 1.32 |
| Respiratory Hospital Admissions | Stratified by sex - Man | 4-days moving average | 112496 | 77 | 1.23 | 1.15 | 1.30 |
| Respiratory Hospital Admissions | Stratified by sex - Man | 5-days moving average | 112482 | 91 | 1.23 | 1.16 | 1.30 |
| Respiratory Hospital Admissions | Stratified by sex - Woman | 1-day moving average | 100693 | 64 | 1.29 | 1.19 | 1.39 |
| Respiratory Hospital Admissions | Stratified by sex - Woman | 2-days moving average | 100700 | 57 | 1.28 | 1.18 | 1.38 |
| Respiratory Hospital Admissions | Stratified by sex - Woman | 3-days moving average | 100698 | 59 | 1.21 | 1.10 | 1.33 |
| Respiratory Hospital Admissions | Stratified by sex - Woman | 4-days moving average | 100686 | 71 | 1.18 | 1.09 | 1.28 |
| Respiratory Hospital Admissions | Stratified by sex - Woman | 5-days moving average | 100677 | 80 | 1.22 | 1.13 | 1.31 |
| Respiratory Hospital Admissions | Stratified by age (0-5 years old) | 1-day moving average | 41129 | 16 | 1.98 | 0.87 | 3.08 |
| Respiratory Hospital Admissions | Stratified by age (0-5 years old) | 2-days moving average | 41121 | 24 | NA | NA | NA |
| Respiratory Hospital Admissions | Stratified by age (0-5 years old) | 3-days moving average | 41128 | 17 | NA | NA | NA |
| Respiratory Hospital Admissions | Stratified by age (0-5 years old) | 4-days moving average | 41121 | 24 | 2.12 | 1.51 | 2.73 |
| Respiratory Hospital Admissions | Stratified by age (0-5 years old) | 5-days moving average | 41113 | 32 | 1.72 | 1.35 | 2.09 |
| Respiratory Hospital Admissions | Stratified by age (35-64 years old) | 1-day moving average | NA | NA | NA | NA | NA |
| Respiratory Hospital Admissions | Stratified by age (35-64 years old) | 2-days moving average | 52430 | 30 | 1.25 | 1.08 | 1.42 |
| Respiratory Hospital Admissions | Stratified by age (35-64 years old) | 3-days moving average | 52431 | 29 | 1.88 | 1.38 | 2.39 |
| Respiratory Hospital Admissions | Stratified by age (35-64 years old) | 4-days moving average | 52428 | 32 | 1.44 | 1.19 | 1.68 |
| Respiratory Hospital Admissions | Stratified by age (35-64 years old) | 5-days moving average | 52421 | 39 | 1.35 | 1.15 | 1.55 |
| Respiratory Hospital Admissions | Stratified by age (> 64 years old) | 1-day moving average | 72650 | 50 | 1.23 | 1.10 | 1.35 |
| Respiratory Hospital Admissions | Stratified by age (> 64 years old) | 2-days moving average | 72661 | 39 | 1.33 | 1.19 | 1.47 |
| Respiratory Hospital Admissions | Stratified by age (> 64 years old) | 3-days moving average | 72654 | 46 | 1.18 | 1.08 | 1.28 |
| Respiratory Hospital Admissions | Stratified by age (> 64 years old) | 4-days moving average | 72645 | 55 | 1.20 | 1.11 | 1.28 |
| Respiratory Hospital Admissions | Stratified by age (> 64 years old) | 5-days moving average | 72639 | 61 | 1.20 | 1.11 | 1.30 |
| Respiratory Hospital Admissions | Primary model excluding the other pollutants (CO, NO2, and O3) | 1-day moving average | 213206 | 124 | 1.30 | 1.27 | 1.34 |
| Respiratory Hospital Admissions | Primary model excluding the other pollutants (CO, NO2, and O3) | 2-days moving average | 213216 | 114 | 1.30 | 1.26 | 1.34 |
| Respiratory Hospital Admissions | Primary model excluding the other pollutants (CO, NO2, and O3) | 3-days moving average | 213208 | 122 | 1.33 | 1.29 | 1.37 |
| Respiratory Hospital Admissions | Primary model excluding the other pollutants (CO, NO2, and O3) | 4-days moving average | 213187 | 143 | 1.30 | 1.27 | 1.34 |
| Respiratory Hospital Admissions | Primary model excluding the other pollutants (CO, NO2, and O3) | 5-days moving average | 213161 | 169 | 1.30 | 1.26 | 1.33 |
| Respiratory Hospital Admissions | Primary model excluding race | 1-day moving average | 213206 | 124 | 1.16 | 1.10 | 1.22 |
| Respiratory Hospital Admissions | Primary model excluding race | 2-days moving average | 213216 | 114 | 1.22 | 1.17 | 1.28 |
| Respiratory Hospital Admissions | Primary model excluding race | 3-days moving average | 213208 | 122 | 1.21 | 1.16 | 1.27 |
| Respiratory Hospital Admissions | Primary model excluding race | 4-days moving average | 213187 | 143 | 1.22 | 1.16 | 1.27 |
| Respiratory Hospital Admissions | Primary model excluding race | 5-days moving average | 213161 | 169 | 1.21 | 1.15 | 1.26 |
| Respiratory Hospital Admissions | Primary model excluding state and lat/long | 1-day moving average | 213206 | 124 | 1.17 | 1.11 | 1.23 |
| Respiratory Hospital Admissions | Primary model excluding state and lat/long | 2-days moving average | 213216 | 114 | 1.22 | 1.16 | 1.27 |
| Respiratory Hospital Admissions | Primary model excluding state and lat/long | 3-days moving average | 213208 | 122 | 1.21 | 1.16 | 1.27 |
| Respiratory Hospital Admissions | Primary model excluding state and lat/long | 4-days moving average | 213187 | 143 | 1.21 | 1.16 | 1.26 |
| Respiratory Hospital Admissions | Primary model excluding state and lat/long | 5-days moving average | 213161 | 169 | 1.21 | 1.15 | 1.26 |
