# Supplementary material for: Health impacts of wildfire-related air pollution in Brazil: a nationwide study of more than 2 million hospital admissions between 2008 and 2018
Source: Nat Commun. 2021 Nov 12;12:6555. doi: 10.1038/s41467-021-26822-7 (PMC8589982; doi:10.1038/s41467-021-26822-7)
Supplement: Supplementary file 2 — Reporting Summary [file 41467_2021_26822_MOESM2_ESM.pdf]

nature research

Corresponding author(s): Weeberb J Requia

Last updated by author(s): Jun 29, 2021

Reporting Summary

Nature Research wishes to improve the reproducibility of the work that we publish. This form provides structure for consistency and transparency in reporting. For further information on Nature Research policies, see our Editorial Policies and the Editorial Policy Checklist.

Statistics

For all statistical analyses, confirm that the following items are present in the figure legend, table legend, main text, or Methods section.

n/a	Confirmed	
☐	☒	The exact sample size (n) for each experimental group/condition, given as a discrete number and unit of measurement
☐	☒	A statement on whether measurements were taken from distinct samples or whether the same sample was measured repeatedly
☒	☐	The statistical test(s) used AND whether they are one- or two-sided<br>*Only common tests should be described solely by name; describe more complex techniques in the Methods section.*
☐	☒	A description of all covariates tested
☐	☒	A description of any assumptions or corrections, such as tests of normality and adjustment for multiple comparisons
☐	☒	A full description of the statistical parameters including central tendency (e.g. means) or other basic estimates (e.g. regression coefficient) AND variation (e.g. standard deviation) or associated estimates of uncertainty (e.g. confidence intervals)
☐	☒	For null hypothesis testing, the test statistic (e.g. F, t, r) with confidence intervals, effect sizes, degrees of freedom and P value noted<br>*Give P values as exact values whenever suitable.*
☒	☐	For Bayesian analysis, information on the choice of priors and Markov chain Monte Carlo settings
☒	☐	For hierarchical and complex designs, identification of the appropriate level for tests and full reporting of outcomes
☒	☐	Estimates of effect sizes (e.g. Cohen's d, Pearson's r), indicating how they were calculated

Our web collection on statistics for biologists contains articles on many of the points above.

Software and code

Policy information about availability of computer code

Data collection	Data collection was performed in R (version 4.0.0)
Data analysis	All statistical analyses were performed in R, using the statistical package Survival ("clogit" function), version 3.2-11

For manuscripts utilizing custom algorithms or software that are central to the research but not yet described in published literature, software must be made available to editors and reviewers. We strongly encourage code deposition in a community repository (e.g. GitHub). See the Nature Research guidelines for submitting code & software for further information.

Data

Policy information about availability of data

All manuscripts must include a data availability statement. This statement should provide the following information, where applicable:
- Accession codes, unique identifiers, or web links for publicly available datasets
- A list of figures that have associated raw data
- A description of any restrictions on data availability

The health data that support the findings of this study are available from the authors on reasonable request, see author contributions for specific data sets. This is because the health data used in our analysis contains confidentiality issues (we accessed personal data). Regarding the exposure variables (air pollution, weather, wildfire), they are available in publicly specific repositories. We mentioned all these repositories in the manuscript.

April 2020

Field-specific reporting

Please select the one below that is the best fit for your research. If you are not sure, read the appropriate sections before making your selection.

☐ Life sciences ☐ Behavioural & social sciences ☒ Ecological, evolutionary & environmental sciences

For a reference copy of the document with all sections, see nature.com/documents/nr-reporting-summary-flat.pdf

Ecological, evolutionary & environmental sciences study design

All studies must disclose on these points even when the disclosure is negative.

Study description	We applied a time-stratified case-crossover study design using conditional logistic regression models.
Research sample	The hospital admission data were provided by the Ministry of Health in Brazil. The data encompass individual records of hospital admissions in Brazil between 2008 and 2018. This data is based on filling out of all Hospital Admission Authorization Forms (HAAFs). During the period between 2008 and 2018, there were 123,479,353 HAAFs in Brazil. In this study we examined only respiratory (ICD-10 codes J00-J99) and cardiovascular (ICD-10 codes I00-I99) diseases. After applying this filter, our study population included 2,044,038 hospital admissions for cardiorespiratory diseases in Brazil between 2008 and 2018
Sampling strategy	The hospital admission data were provided by the Ministry of Health in Brazil. The data encompass individual records of hospital admissions in Brazil between 2008 and 2018. This data is based on filling out of all Hospital Admission Authorization Forms (HAAFs). During the period between 2008 and 2018, there were 123,479,353 HAAFs in Brazil. Hospital admission information included principal diagnosis according to the International Classification of Diseases, version 10 (ICD-10) codes. Based on on this information, we selected only respiratory (ICD-10 codes J00-J99) and cardiovascular (ICD-10 codes I00-I99) diseases. This selection resulted in a study population of 2,044,038 hospital admissions for cardiorespiratory diseases in Brazil between 2008 and 2018. Among those, 50.2 % are respiratory diseases and 49.8 % circulatory diseases.
Data collection	The data were provided by the Ministry of Health in Brazil.
Timing and spatial scale	The data provided by the Ministry of Health in Brazil is based on the municipality scale in Brazil for the period between 2008 and 2018. In our analyses, we used these original timing and spatial scale.
Data exclusions	We excluded all hospital admissions records that were not identified as Irespiratory (ICD-10 codes J00-J99) and cardiovascular (ICD-10 codes I00-I99) diseases.
Reproducibility	The analyzes were repeated 5 times in different computers by different researchers. All attempts at replication were successful.
Randomization	The randomization is not relevant to our study. Based on the characteristics of the exposure variables, we were able to perform an ecological study
Blinding	Given that we performed an ecological study, blinding was not possible.

Did the study involve field work? ☐ Yes ☒ No

Reporting for specific materials, systems and methods

We require information from authors about some types of materials, experimental systems and methods used in many studies. Here, indicate whether each material, system or method listed is relevant to your study. If you are not sure if a list item applies to your research, read the appropriate section before selecting a response.

Materials & experimental systems

n/a	Involved in the study	
☒	☐	Antibodies
☒	☐	Eukaryotic cell lines
☒	☐	Palaeontology and archaeology
☒	☐	Animals and other organisms
☐	☒	Human research participants
☒	☐	Clinical data
☒	☐	Dual use research of concern

Methods

n/a	Involved in the study	
☒	☐	ChIP-seq
☒	☐	Flow cytometry
☒	☐	MRI-based neuroimaging

Human research participants

Policy information about studies involving human research participants

Population characteristics	Our study population includes 2,044,038 hospital admissions for cardiorespiratory diseases in Brazil between 2008 and 2018. Among those, 50.2 % are respiratory diseases and 49.8 % circulatory diseases. For respiratory hospital admissions, 52.9% are males, 25.7% are patients aged < 5 years old, 20.8% are patients aged 35-64 years old, and 27.6% aged > 64 years old. For circulatory hospital admissions, 50.4% are males, 0.7% are patients aged < 5 years old, 46.6% are patients aged 35-64 years old, and 45.7% aged > 64 years old.
Recruitment	We did not recruit any individual. All the data were provided by the Ministry of Health in Brazil
Ethics oversight	The Ministry of Health in Brazil

Note that full information on the approval of the study protocol must also be provided in the manuscript.
